# Supplementary material for: ACK1 and BRK non-receptor tyrosine kinase deficiencies are associated with familial systemic lupus and involved in efferocytosis
Source: eLife. 2024 Nov 21;13:RP96085. doi: 10.7554/eLife.96085 (PMC11581429; doi:10.7554/eLife.96085)
Supplement: Figure 2—source data 3. [file elife-96085-fig2-data3.zip › Figure 2-Source Data 3 - Uncropped and labelled gels for Figure 2/Figure 2-Source Data 3 - Uncropped and labelled gels - Related to Figure 2E.pdf]

Full unedited gels for Figure 2E. The red boxes show the image used in the manuscript.

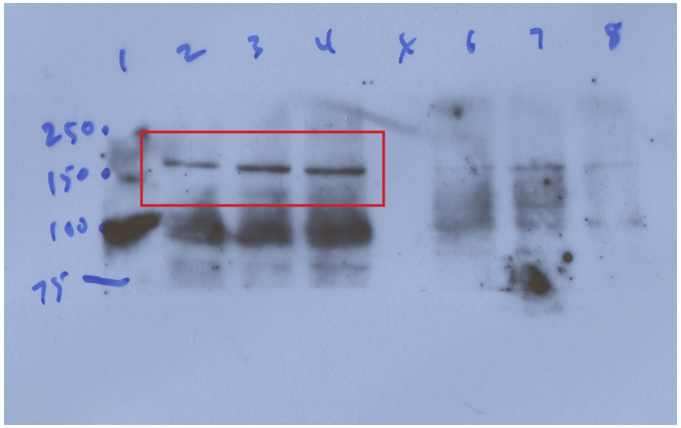

ACK1

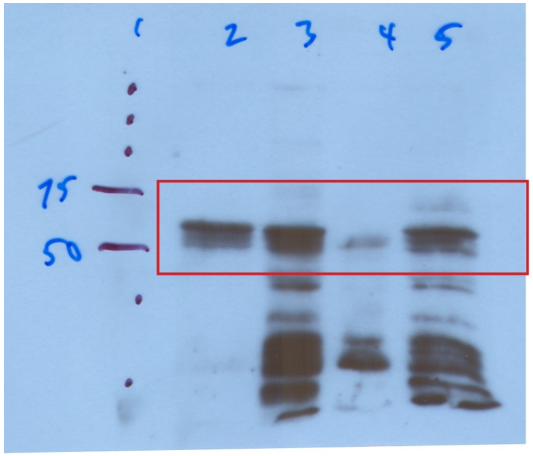

pY342

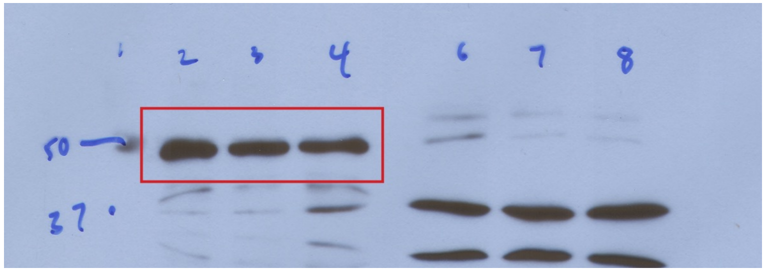

tubulin

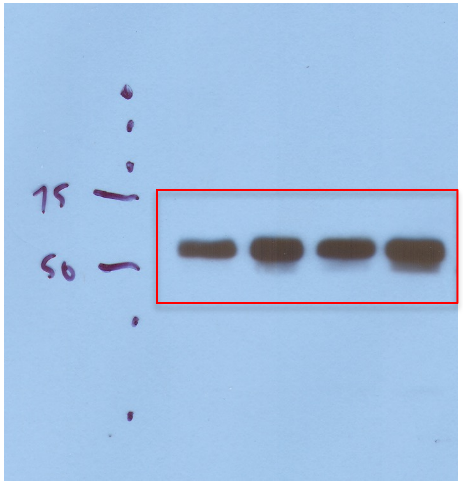

BRK
