# Supplementary material for: ACK1 and BRK non-receptor tyrosine kinase deficiencies are associated with familial systemic lupus and involved in efferocytosis
Source: eLife. 2024 Nov 21;13:RP96085. doi: 10.7554/eLife.96085 (PMC11581429; doi:10.7554/eLife.96085)

Full unedited gels for Figure 2C (top). The red boxes show the images used in the manuscript.

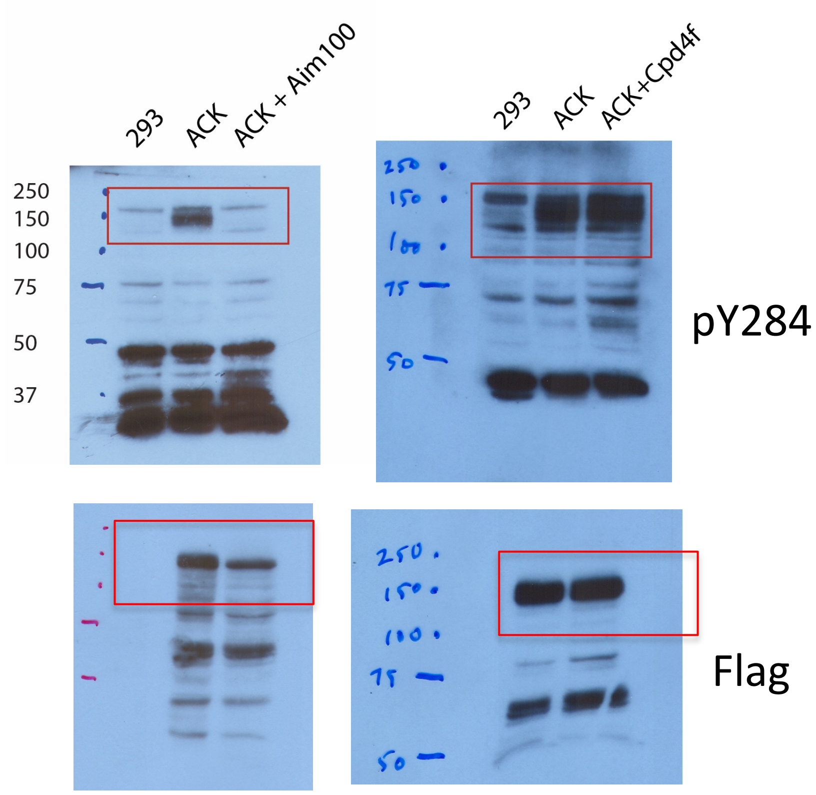

Full unedited gels for Figure 2C (bottom). The red boxes show the images used in the manuscript.

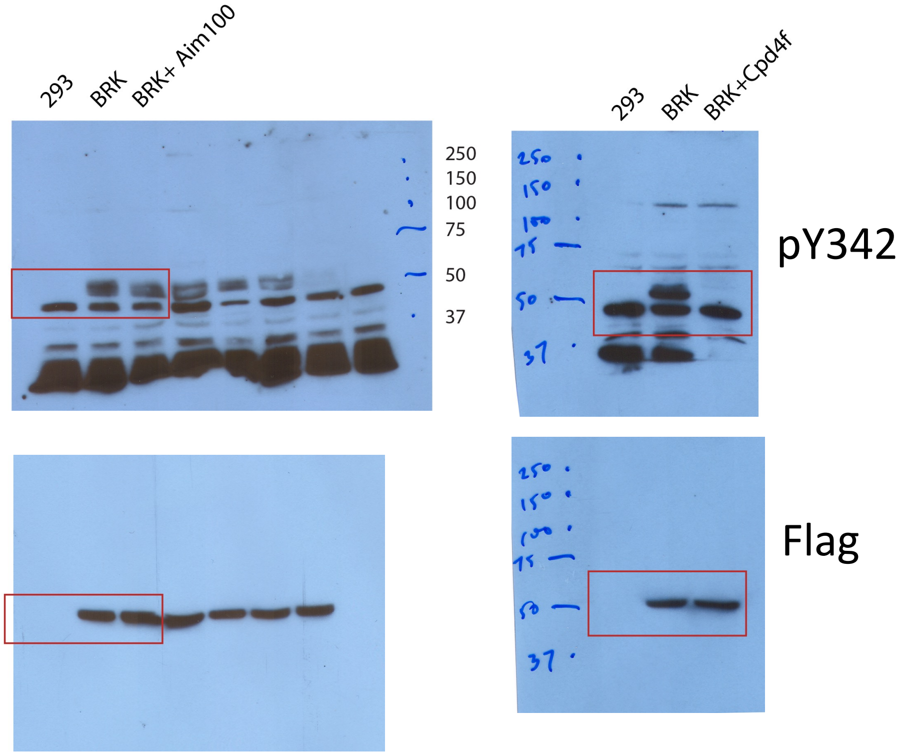

Supplement: Figure 2—source data 3. [file elife-96085-fig2-data3.zip › Figure 2-Source Data 3 - Uncropped and labelled gels for Figure 2/Figure 2-Source Data 3 - Uncropped and labelled gels - Related to Figure 2C.pdf]
