# Supplementary material for: ACK1 and BRK non-receptor tyrosine kinase deficiencies are associated with familial systemic lupus and involved in efferocytosis
Source: eLife. 2024 Nov 21;13:RP96085. doi: 10.7554/eLife.96085 (PMC11581429; doi:10.7554/eLife.96085)
Supplement: Figure 2—source data 3. [file elife-96085-fig2-data3.zip › Figure 2-Source Data 3 - Uncropped and labelled gels for Figure 2/Figure 2-Source Data 3 - Uncropped and labelled gels - Related to Figure 2B.pdf]

Full unedited gels for Figure 2B (left). The red boxes show the images used in the manuscript.

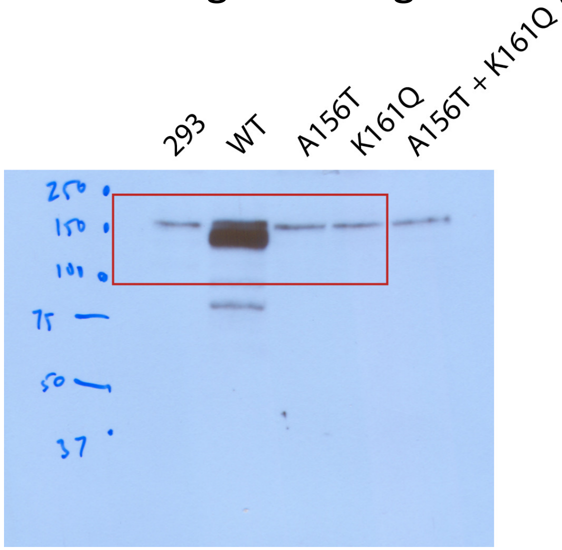

pY284

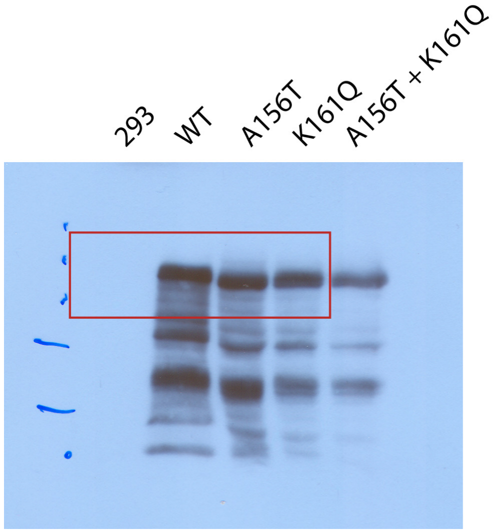

Flag

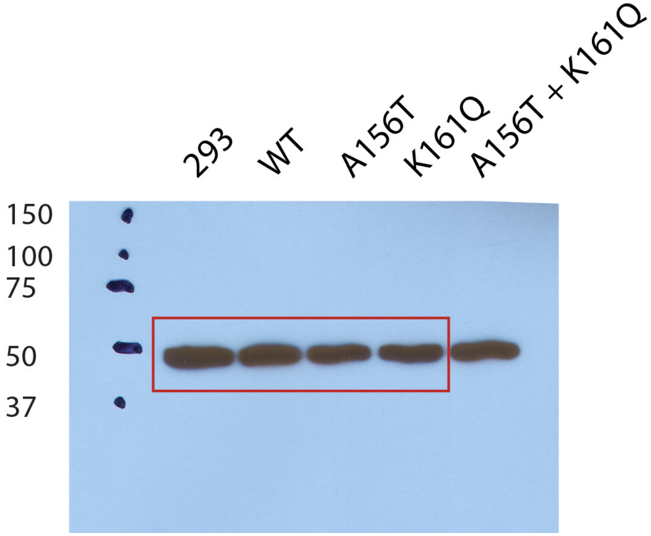

Tubulin

Full unedited gels for Figure 2B (right). The red boxes show the images used in the manuscript.

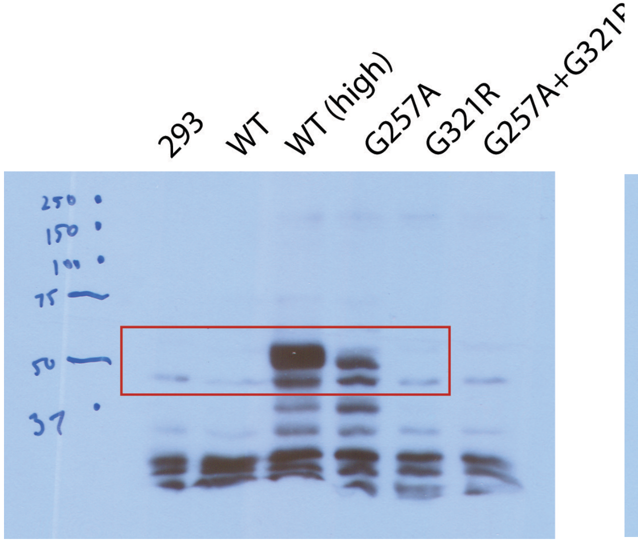

pY342

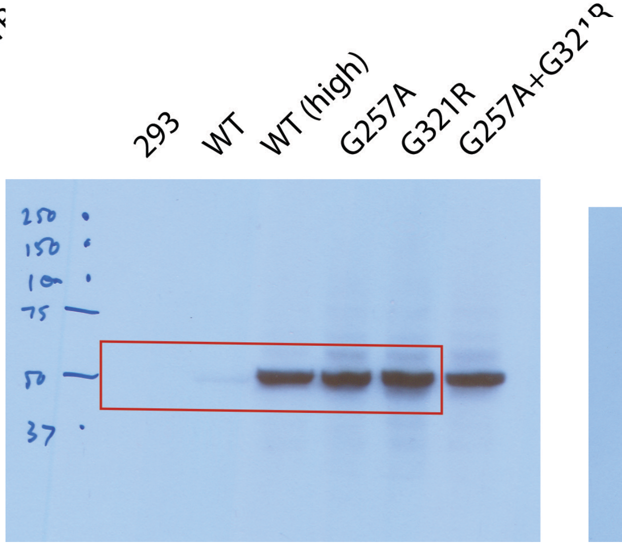

Flag

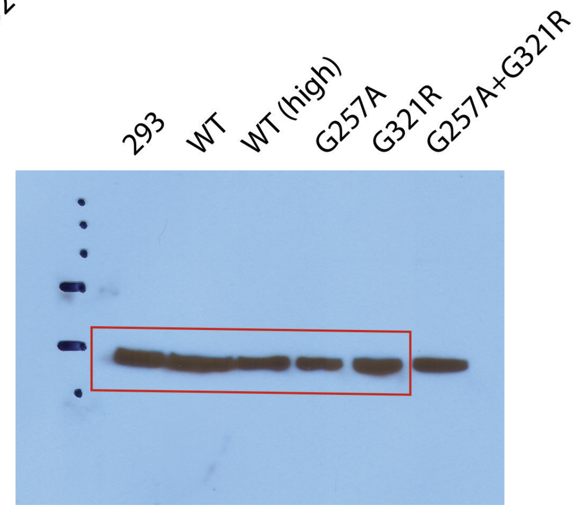

Tubulin
