# Supplementary material for: ACK1 and BRK non-receptor tyrosine kinase deficiencies are associated with familial systemic lupus and involved in efferocytosis
Source: eLife. 2024 Nov 21;13:RP96085. doi: 10.7554/eLife.96085 (PMC11581429; doi:10.7554/eLife.96085)

Full unedited gel for Figure 2A (left). The red box shows the image used in the manuscript.

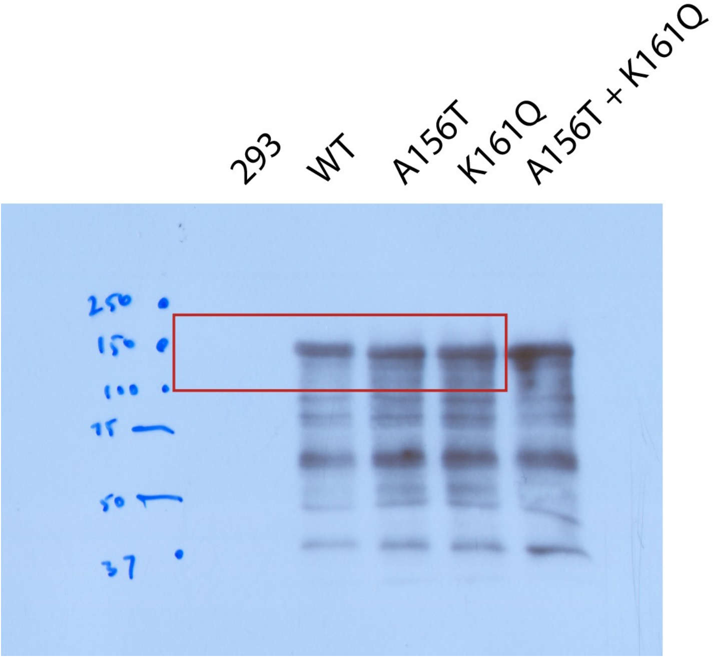

Full unedited gel for Figure 2A (right). The red box shows the image used in the manuscript.

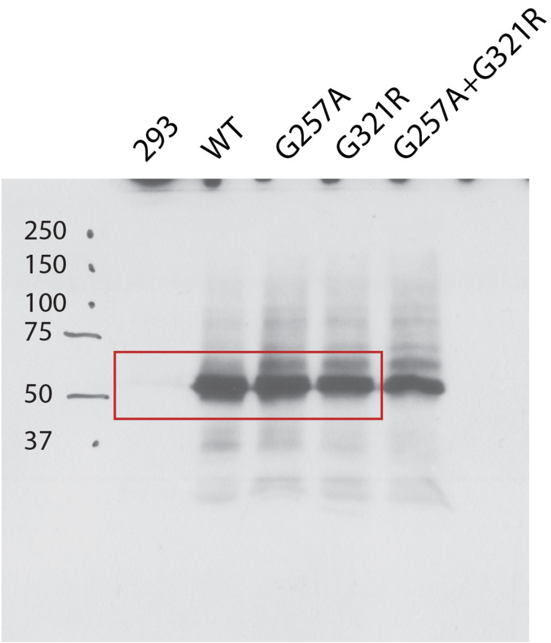

Supplement: Figure 2—source data 3. [file elife-96085-fig2-data3.zip › Figure 2-Source Data 3 - Uncropped and labelled gels for Figure 2/Figure 2-Source Data 3 - Uncropped and labelled gels - Related to Figure 2A.pdf]
