# Supplementary figures and images for: ACK1 and BRK non-receptor tyrosine kinase deficiencies are associated with familial systemic lupus and involved in efferocytosis
Source: eLife. 2024 Nov 21;13:RP96085. doi: 10.7554/eLife.96085 (PMC11581429; doi:10.7554/eLife.96085)

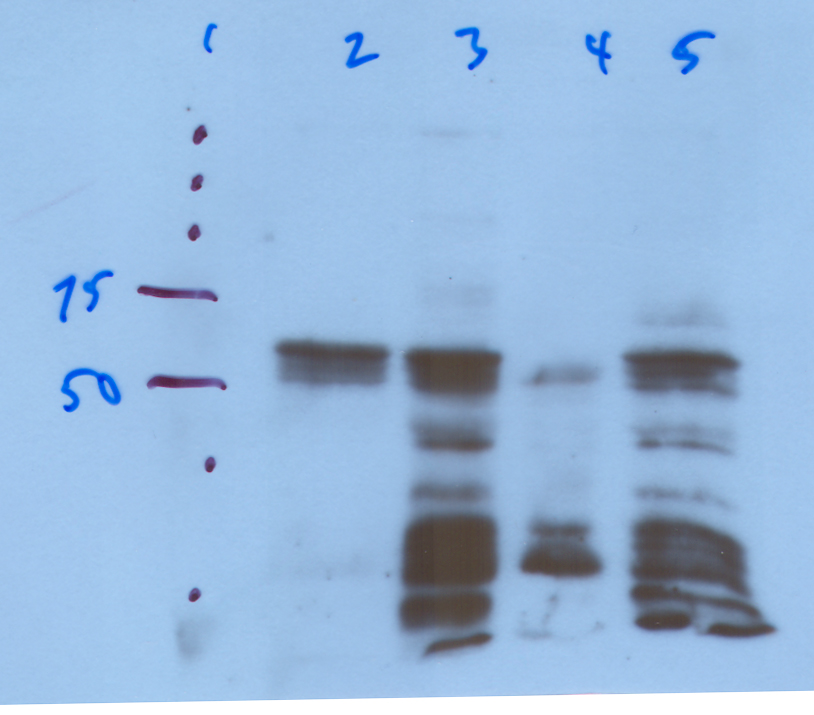

Supplement: Figure 2—source data 4. [file elife-96085-fig2-data4.zip › Figure 2-Source Data 4 - Raw unedited gels for Figure 2/Fig2E_right_top.tiff]

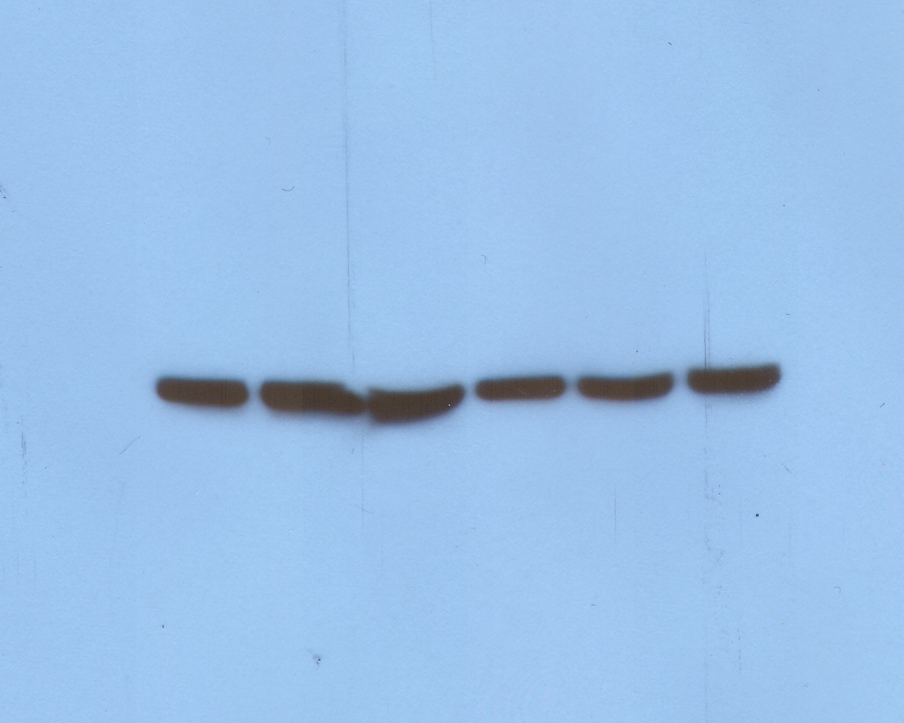

Supplement: Figure 2—source data 4. [file elife-96085-fig2-data4.zip › Figure 2-Source Data 4 - Raw unedited gels for Figure 2/Fig2C_Bottom_Left_FLAG.tiff]

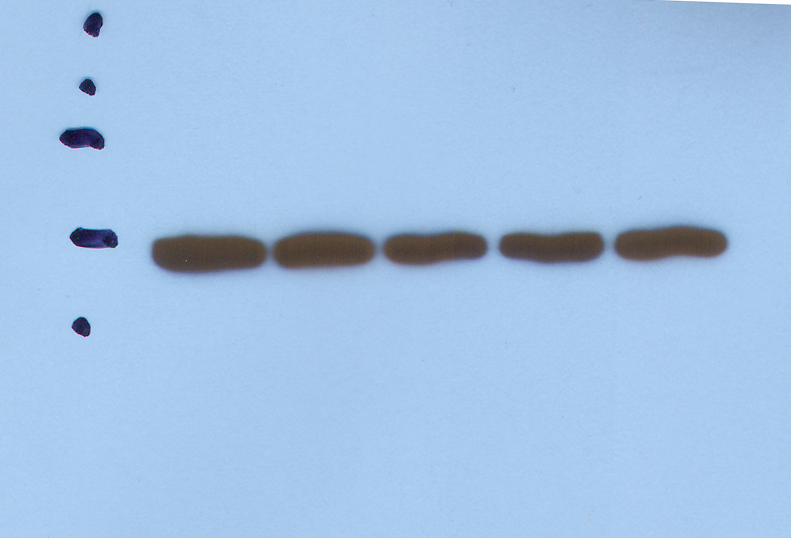

Supplement: Figure 2—source data 4. [file elife-96085-fig2-data4.zip › Figure 2-Source Data 4 - Raw unedited gels for Figure 2/Fig2B_Left_Tubulin.tiff]

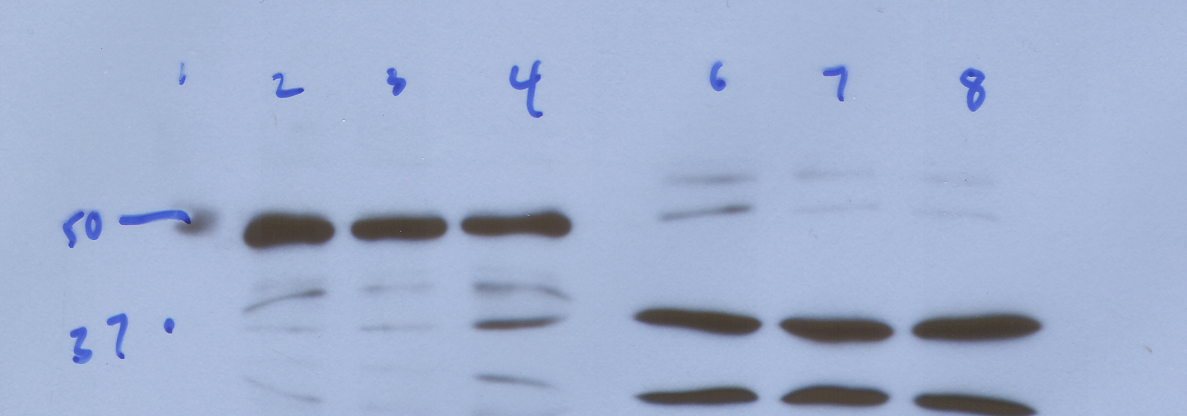

Supplement: Figure 2—source data 4. [file elife-96085-fig2-data4.zip › Figure 2-Source Data 4 - Raw unedited gels for Figure 2/Fig2E_left_bottom.tiff]

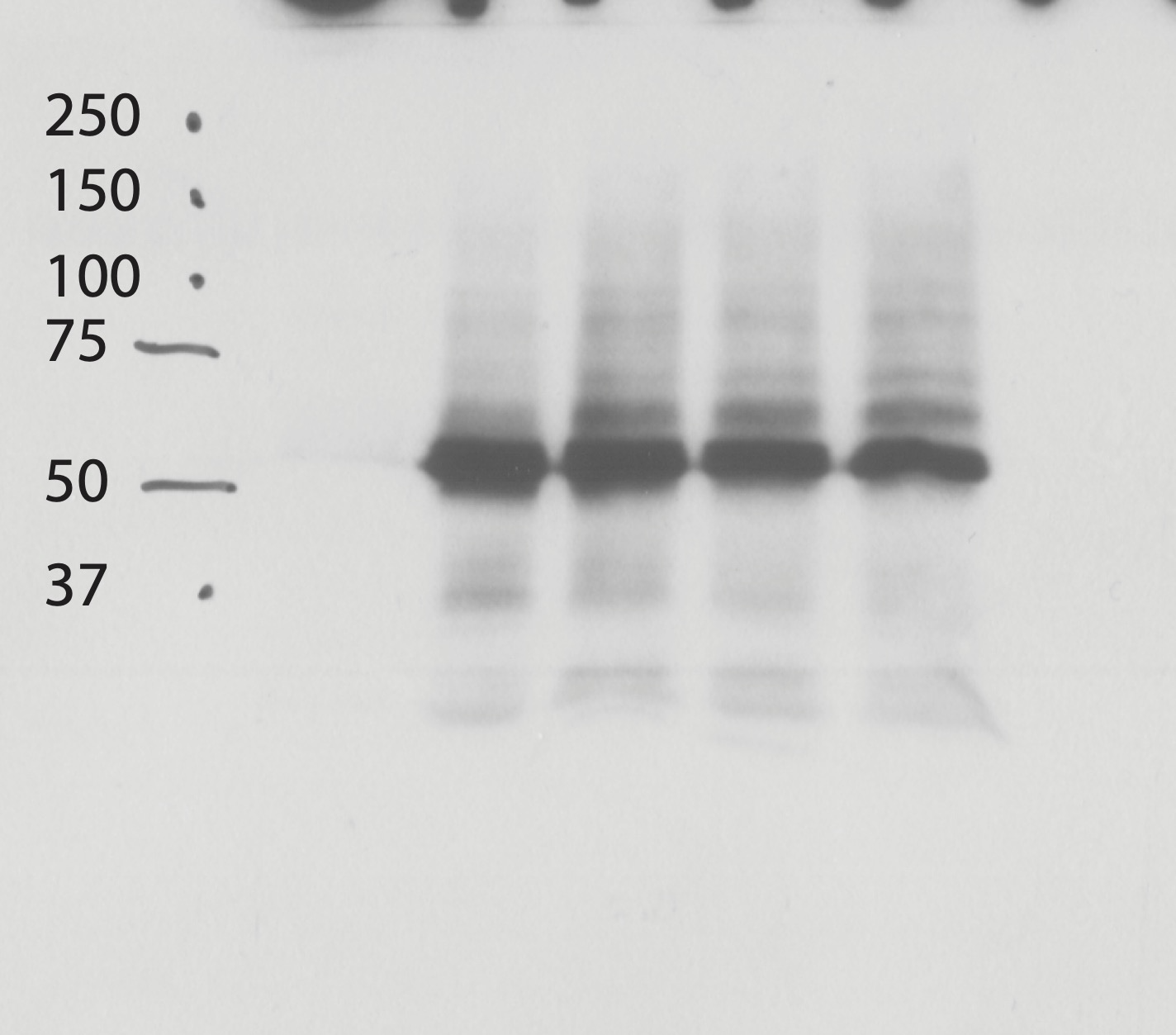

Supplement: Figure 2—source data 4. [file elife-96085-fig2-data4.zip › Figure 2-Source Data 4 - Raw unedited gels for Figure 2/Fig2A_Right.tiff]

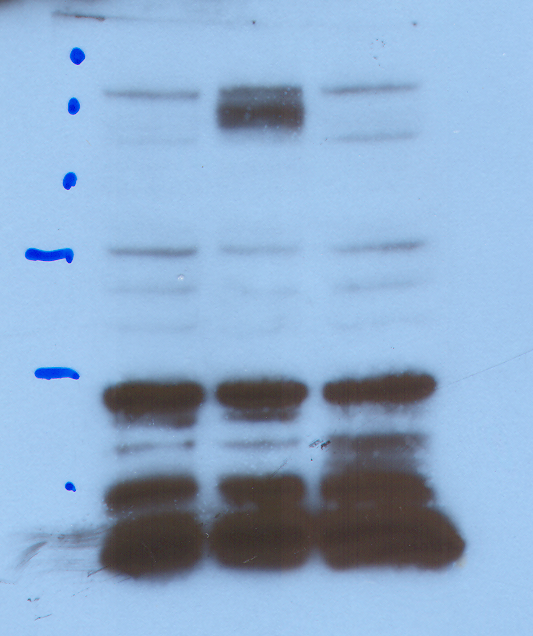

Supplement: Figure 2—source data 4. [file elife-96085-fig2-data4.zip › Figure 2-Source Data 4 - Raw unedited gels for Figure 2/Fig2C_Top_Left_pY284.tif]

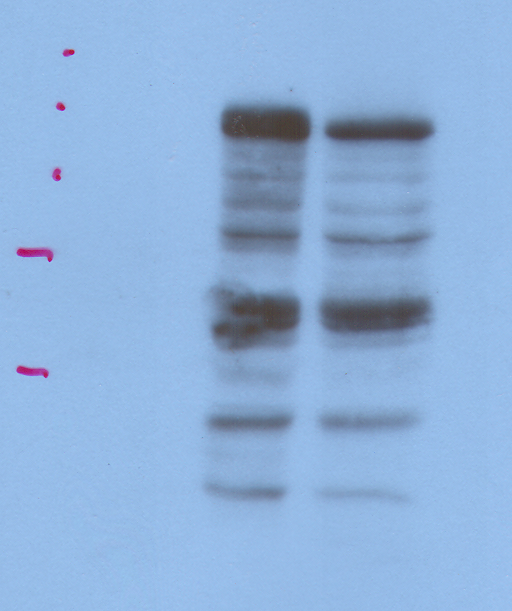

Supplement: Figure 2—source data 4. [file elife-96085-fig2-data4.zip › Figure 2-Source Data 4 - Raw unedited gels for Figure 2/Fig2C_Top_Left_FLAG.tif]

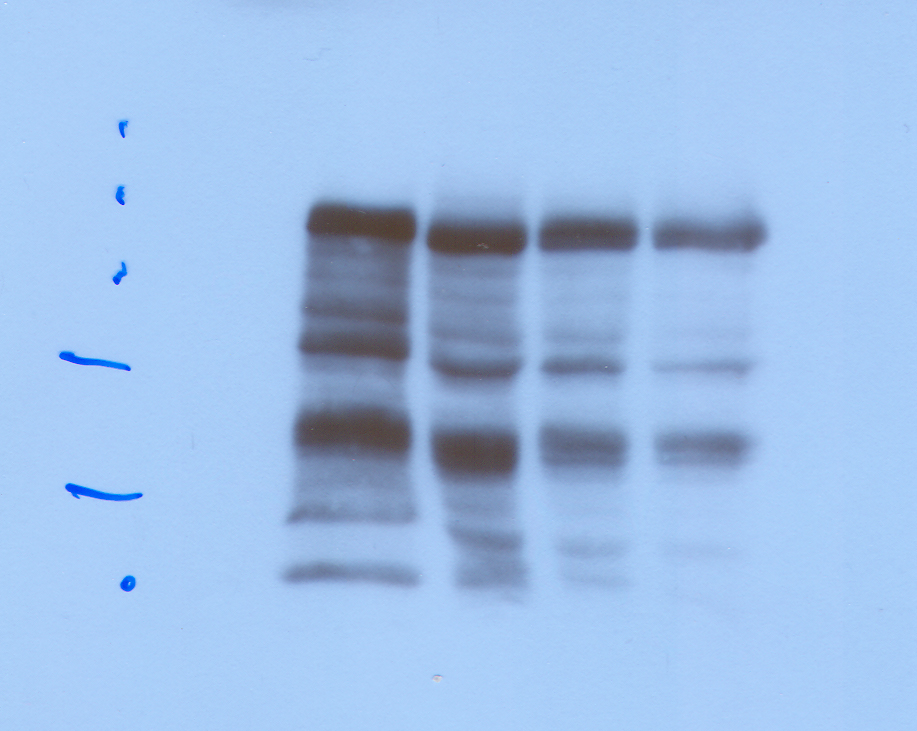

Supplement: Figure 2—source data 4. [file elife-96085-fig2-data4.zip › Figure 2-Source Data 4 - Raw unedited gels for Figure 2/Fig2B_Left_FLAG.tiff]

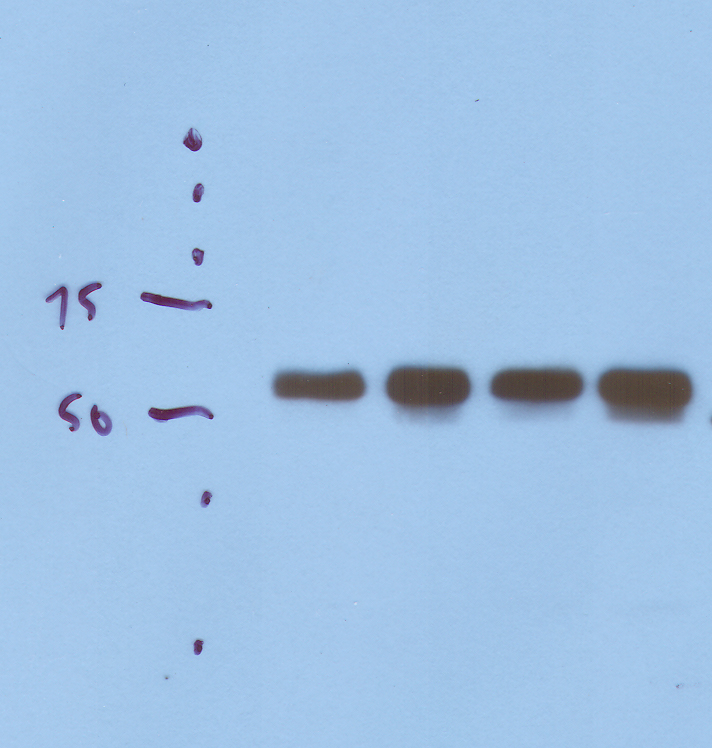

Supplement: Figure 2—source data 4. [file elife-96085-fig2-data4.zip › Figure 2-Source Data 4 - Raw unedited gels for Figure 2/Fig2E_right_bottom.tiff]

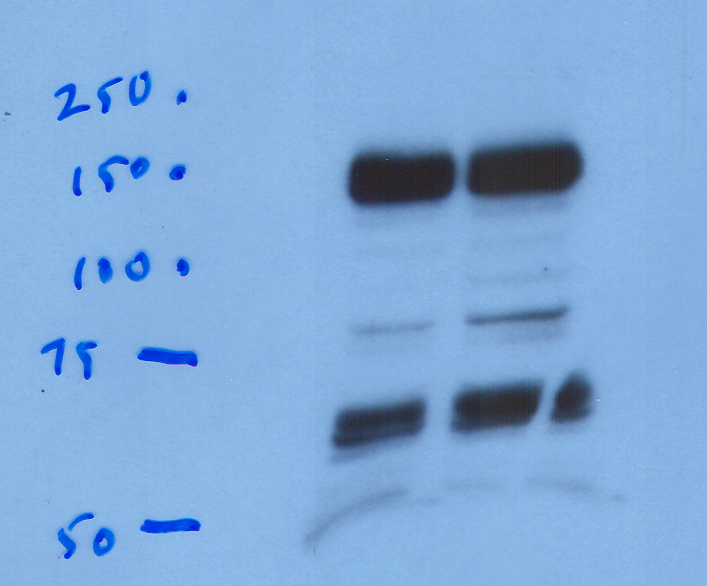

Supplement: Figure 2—source data 4. [file elife-96085-fig2-data4.zip › Figure 2-Source Data 4 - Raw unedited gels for Figure 2/Fig2C_Top_Right_FLAG.tiff]

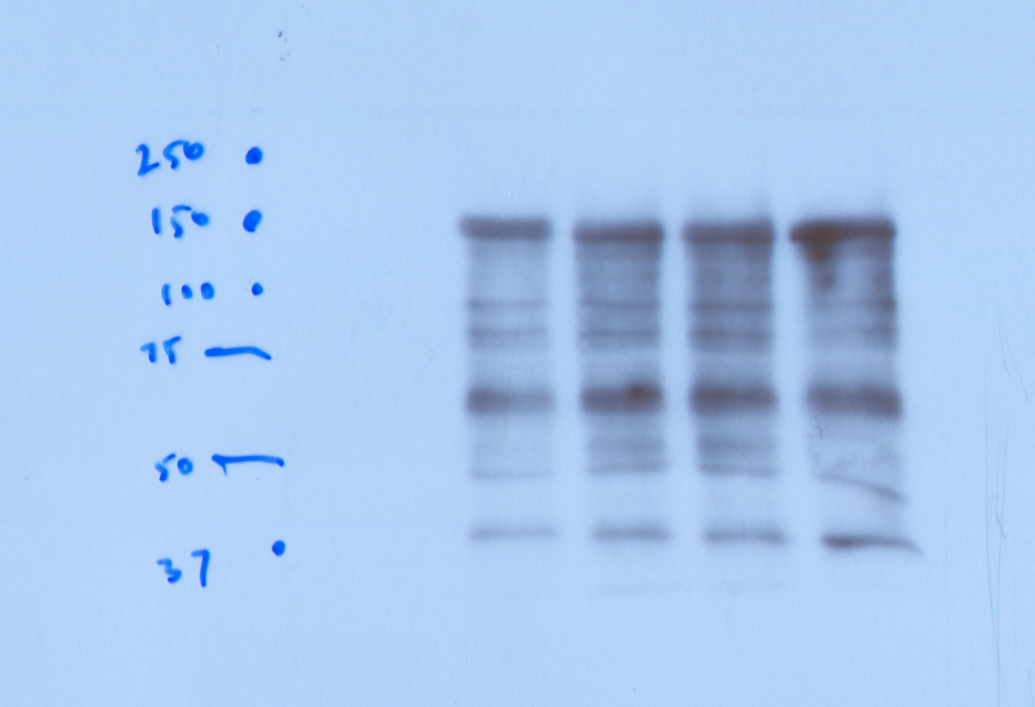

Supplement: Figure 2—source data 4. [file elife-96085-fig2-data4.zip › Figure 2-Source Data 4 - Raw unedited gels for Figure 2/Fig2A_Left.tiff]

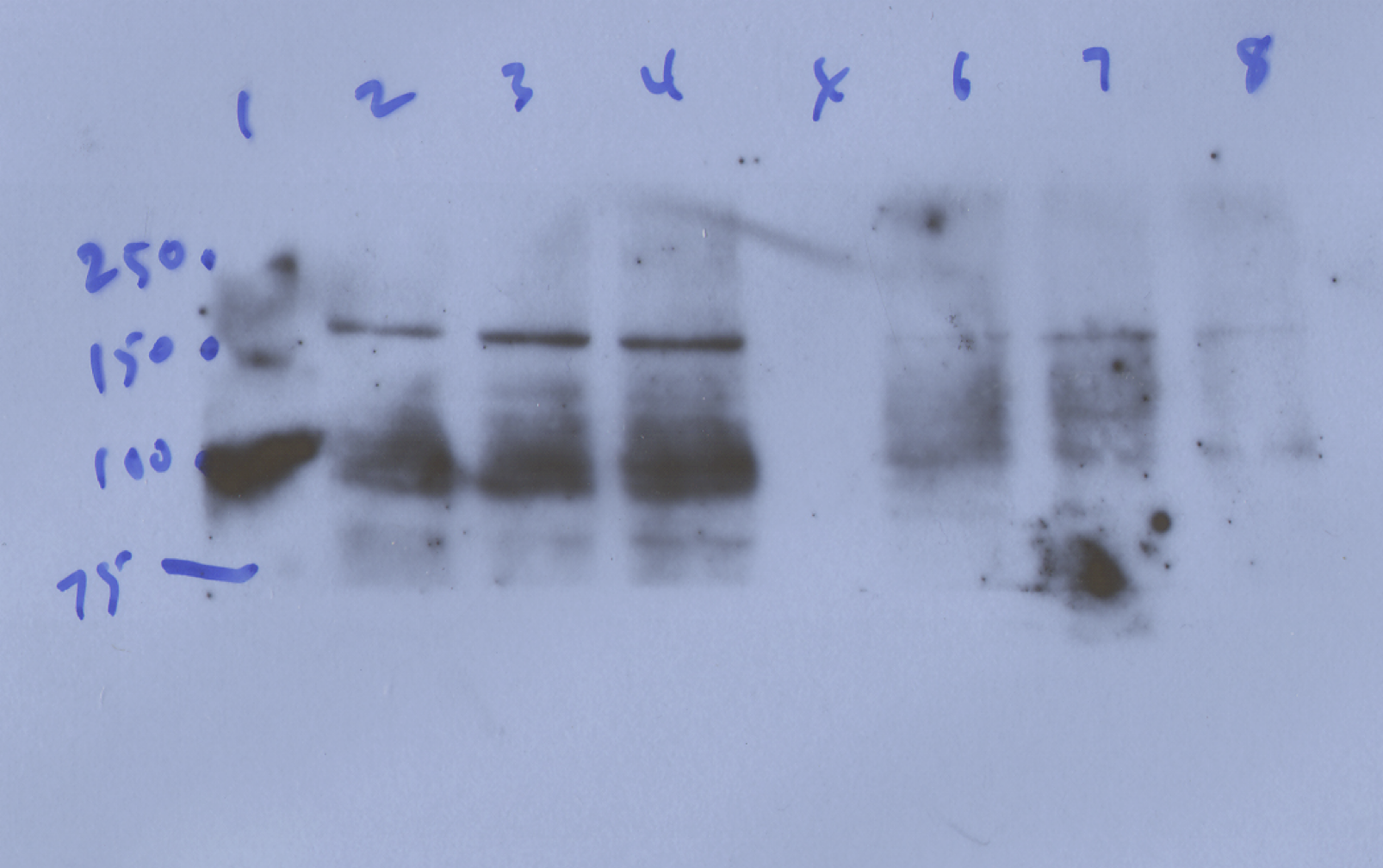

Supplement: Figure 2—source data 4. [file elife-96085-fig2-data4.zip › Figure 2-Source Data 4 - Raw unedited gels for Figure 2/Fig2E_left_top.tiff]

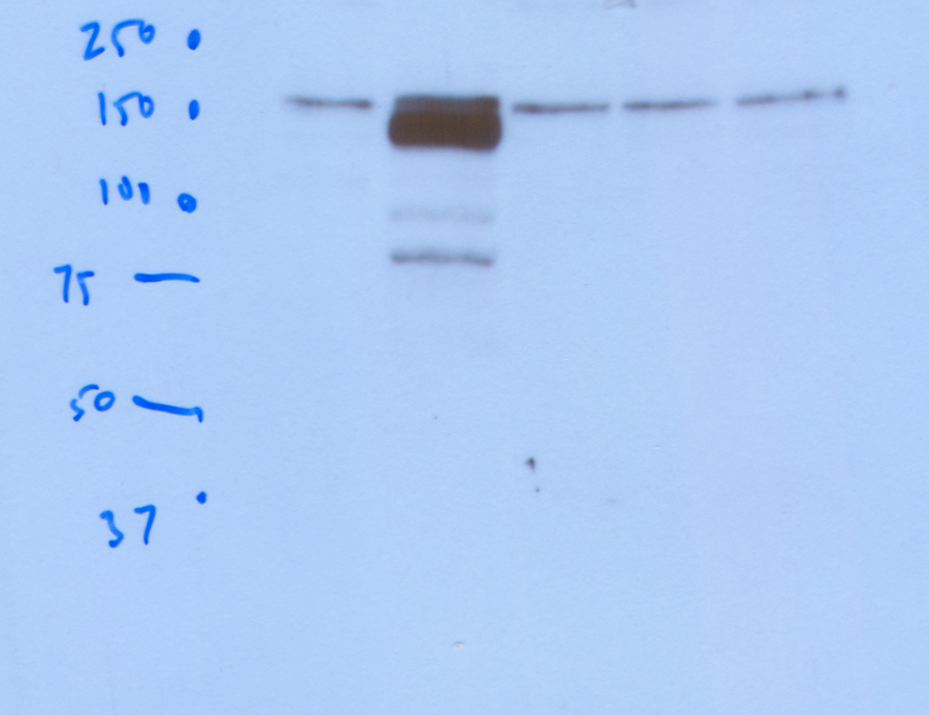

Supplement: Figure 2—source data 4. [file elife-96085-fig2-data4.zip › Figure 2-Source Data 4 - Raw unedited gels for Figure 2/Fig2B_Left_pY284.tiff]

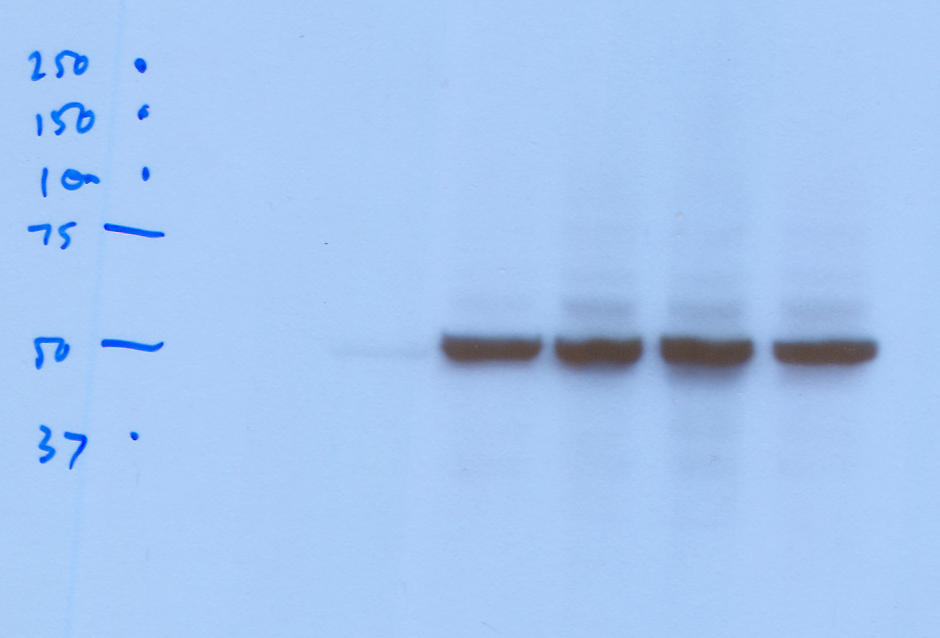

Supplement: Figure 2—source data 4. [file elife-96085-fig2-data4.zip › Figure 2-Source Data 4 - Raw unedited gels for Figure 2/Fig2B_Right_FLAG.tif]

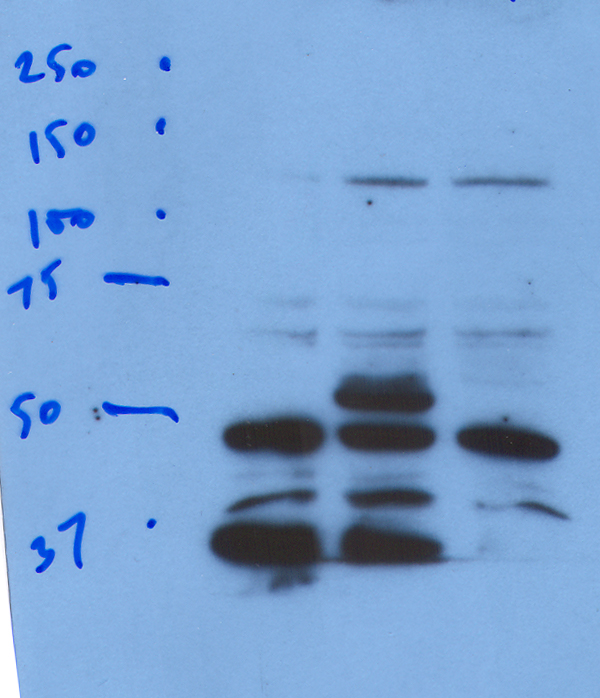

Supplement: Figure 2—source data 4. [file elife-96085-fig2-data4.zip › Figure 2-Source Data 4 - Raw unedited gels for Figure 2/Fig2C_Bottom_Right_pY342.tiff]

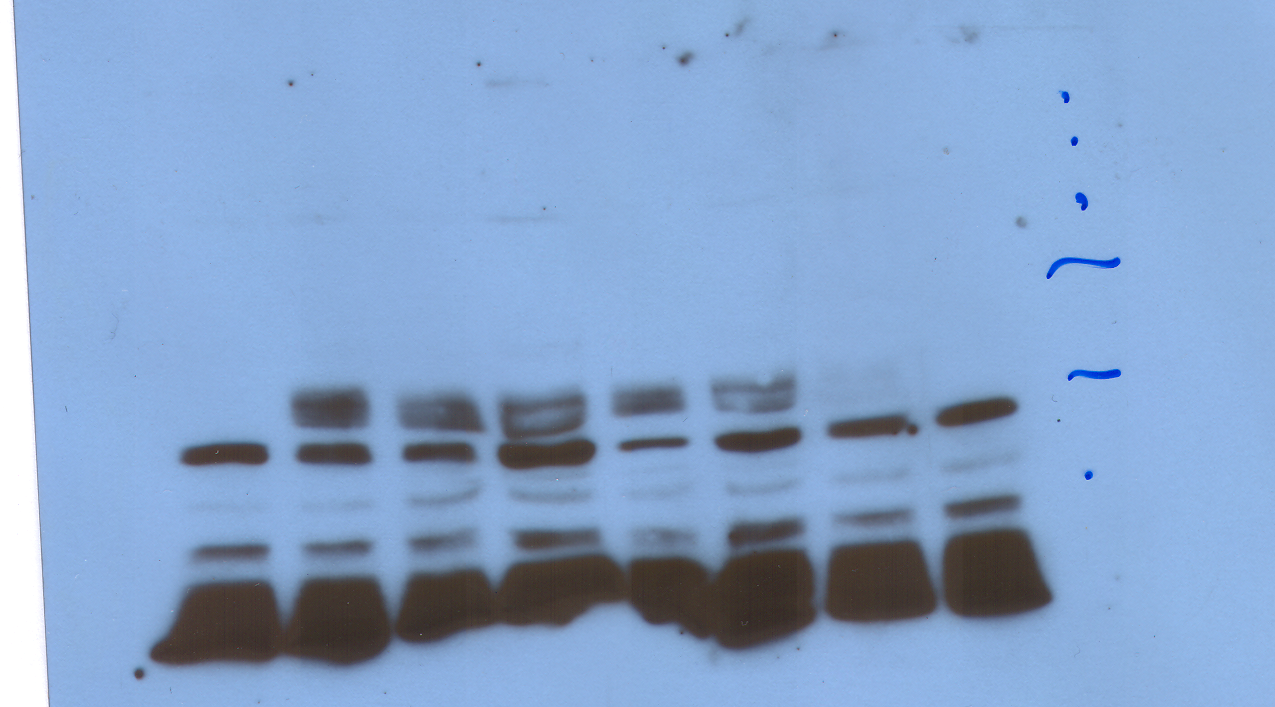

Supplement: Figure 2—source data 4. [file elife-96085-fig2-data4.zip › Figure 2-Source Data 4 - Raw unedited gels for Figure 2/Fig2C_Bottom_Left_pY342.tif]

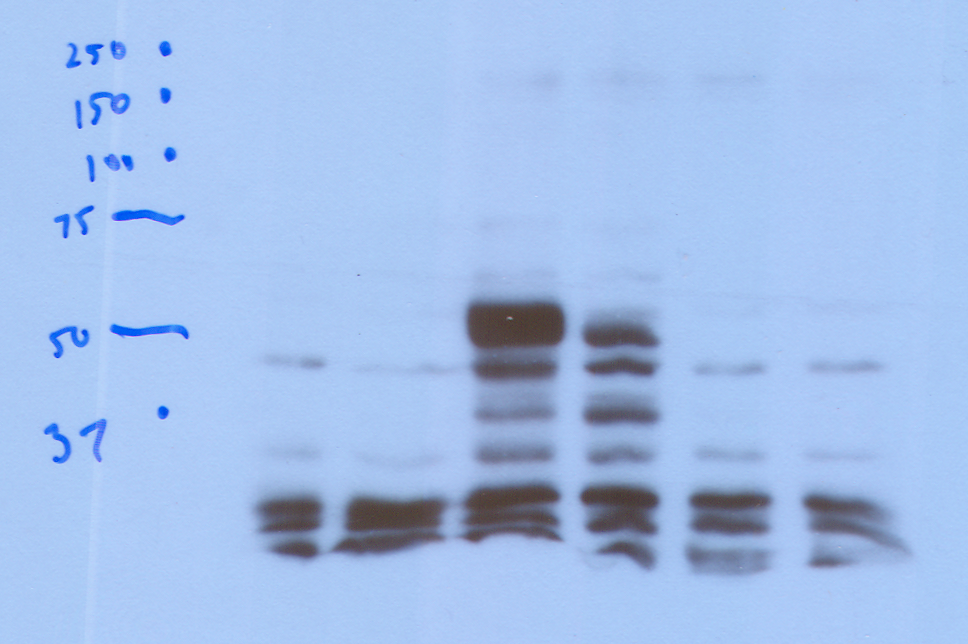

Supplement: Figure 2—source data 4. [file elife-96085-fig2-data4.zip › Figure 2-Source Data 4 - Raw unedited gels for Figure 2/Fig2B_Right_pY342.tif]

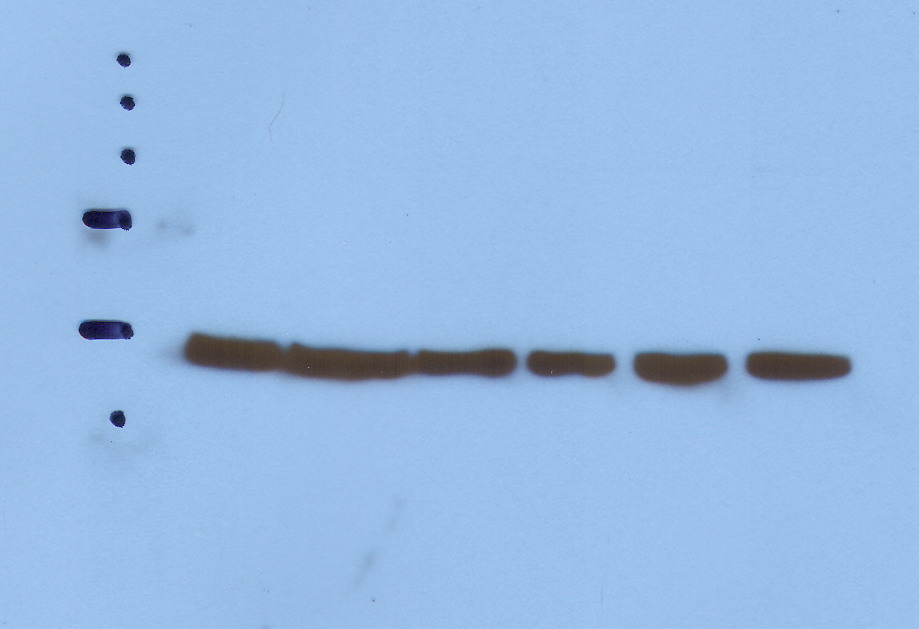

Supplement: Figure 2—source data 4. [file elife-96085-fig2-data4.zip › Figure 2-Source Data 4 - Raw unedited gels for Figure 2/Fig2B_Right_tubulin.tif]

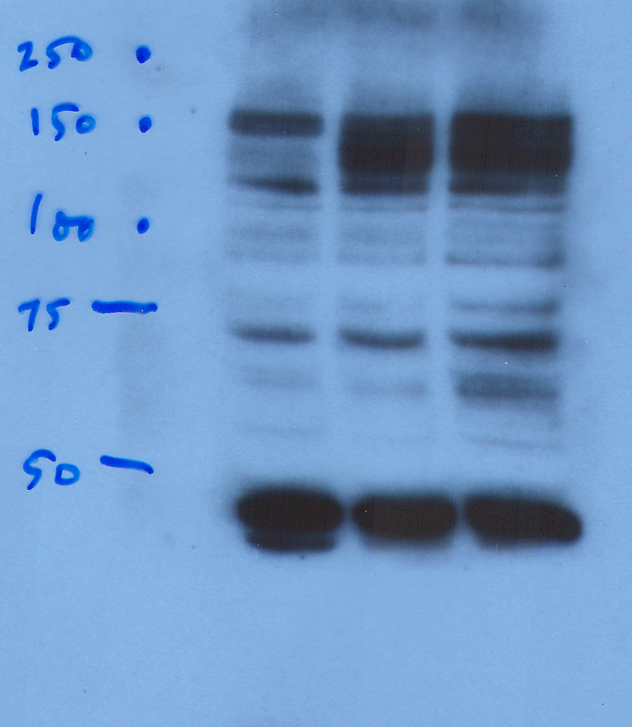

Supplement: Figure 2—source data 4. [file elife-96085-fig2-data4.zip › Figure 2-Source Data 4 - Raw unedited gels for Figure 2/Fig2C_Top_Right_pY284.tif]

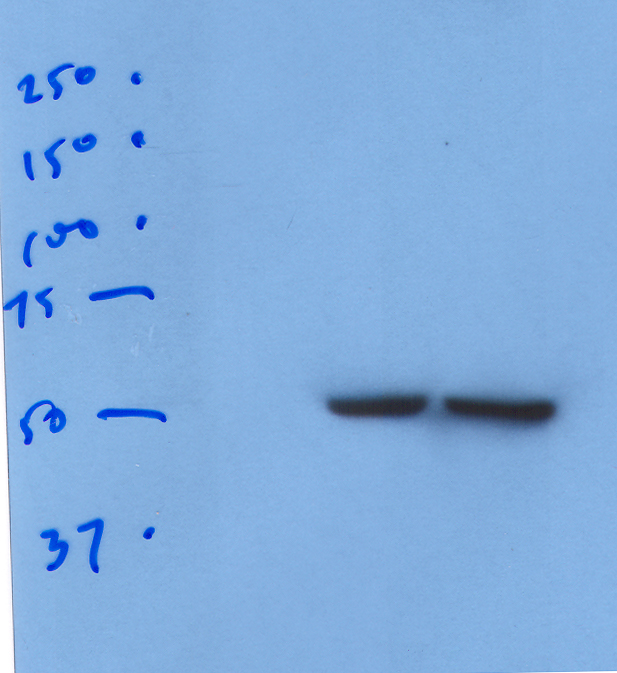

Supplement: Figure 2—source data 4. [file elife-96085-fig2-data4.zip › Figure 2-Source Data 4 - Raw unedited gels for Figure 2/Fig2C_Bottom_Right_FLAG.tiff]

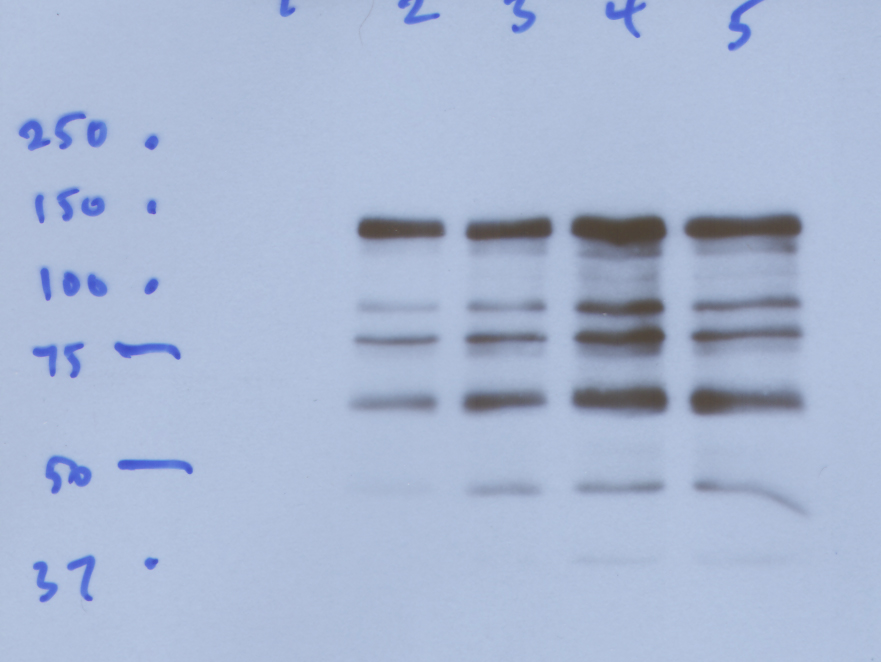

Supplement: Figure 2—figure supplement 2—source data 2. [file elife-96085-fig2-figsupp2-data2.zip › Figure 2-figure supplement 2-Source Data 2 - Raw unedited gels for Figure 2-figure supplement 2/Figure 2-figure supplement 2C_left.tiff]

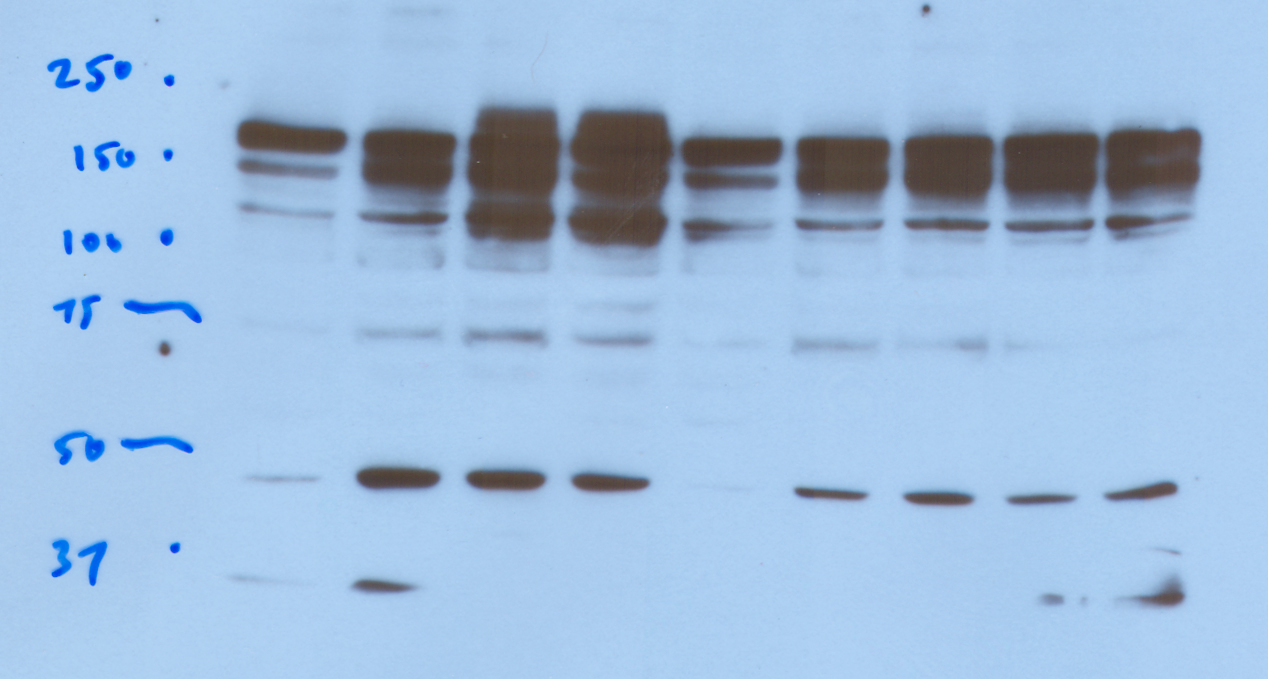

Supplement: Figure 2—figure supplement 2—source data 2. [file elife-96085-fig2-figsupp2-data2.zip › Figure 2-figure supplement 2-Source Data 2 - Raw unedited gels for Figure 2-figure supplement 2/Figure 2-figure supplement 2C_Middle_pY284_dark.tiff]

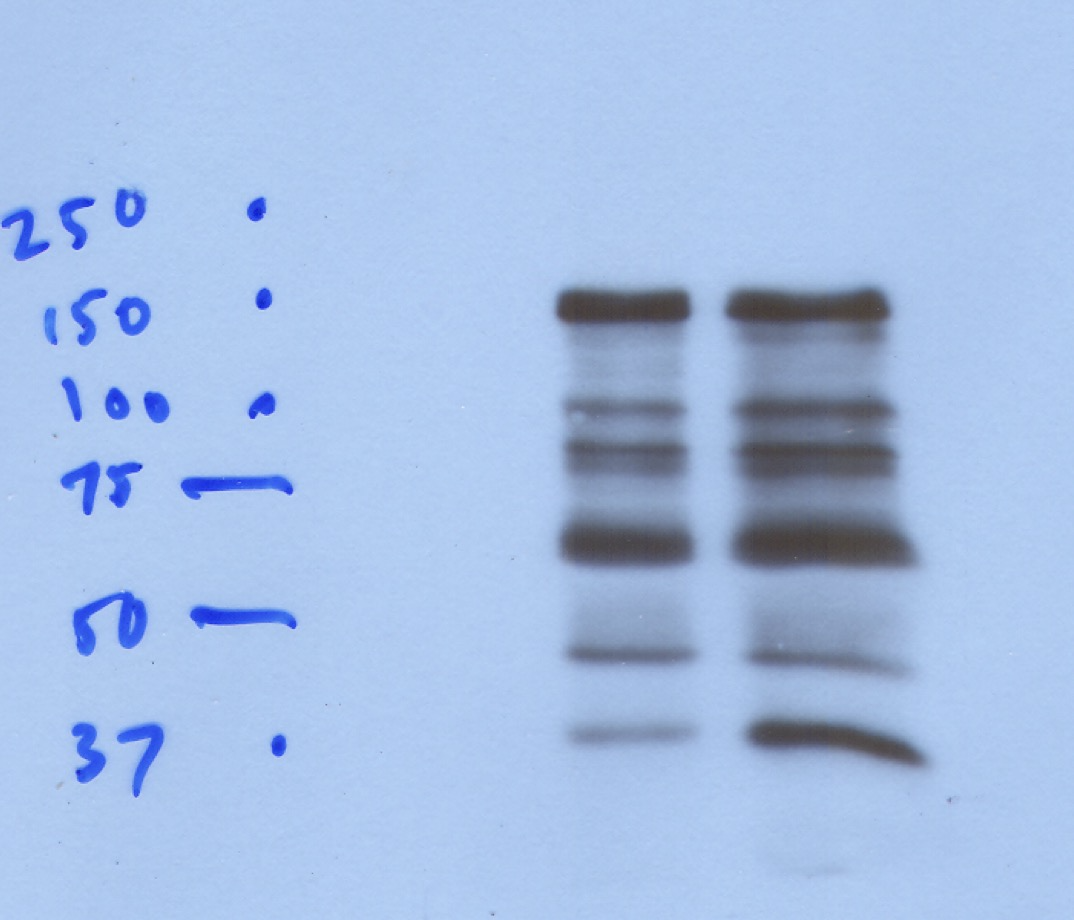

Supplement: Figure 2—figure supplement 2—source data 2. [file elife-96085-fig2-figsupp2-data2.zip › Figure 2-figure supplement 2-Source Data 2 - Raw unedited gels for Figure 2-figure supplement 2/Figure 2-figure supplement 2C_right.tiff]

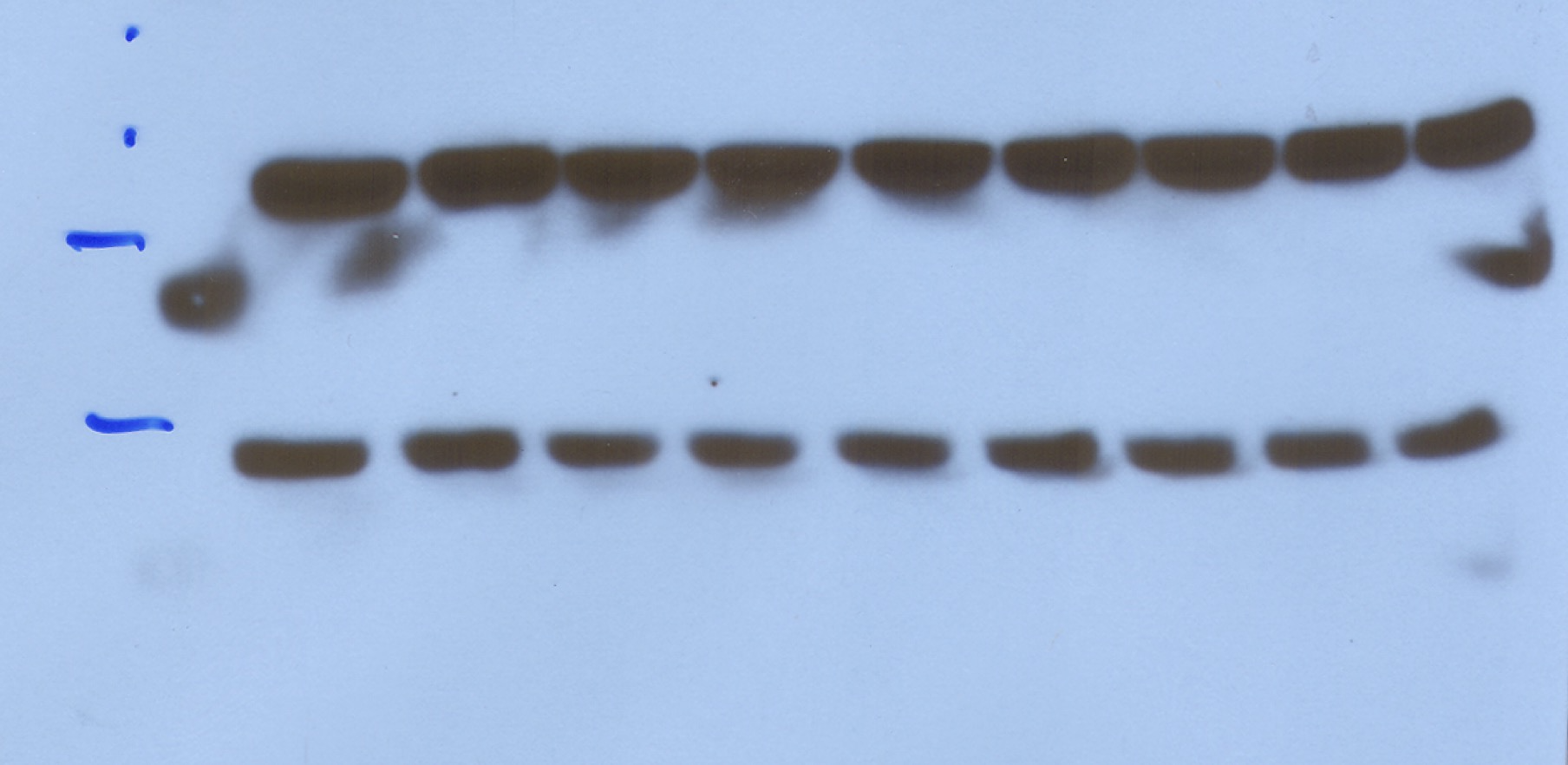

Supplement: Figure 2—figure supplement 2—source data 2. [file elife-96085-fig2-figsupp2-data2.zip › Figure 2-figure supplement 2-Source Data 2 - Raw unedited gels for Figure 2-figure supplement 2/Figure 2-figure supplement 2C_Middle_tubulin.tiff]

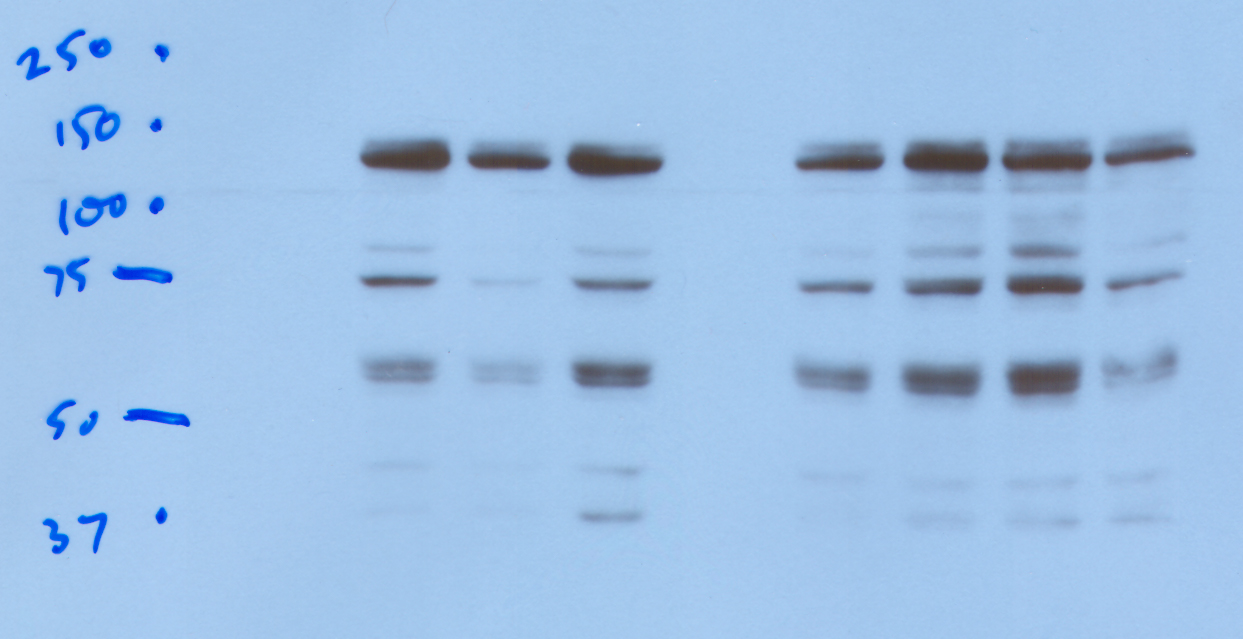

Supplement: Figure 2—figure supplement 2—source data 2. [file elife-96085-fig2-figsupp2-data2.zip › Figure 2-figure supplement 2-Source Data 2 - Raw unedited gels for Figure 2-figure supplement 2/Figure 2-figure supplement 2C_Middle_FLAG.tiff]

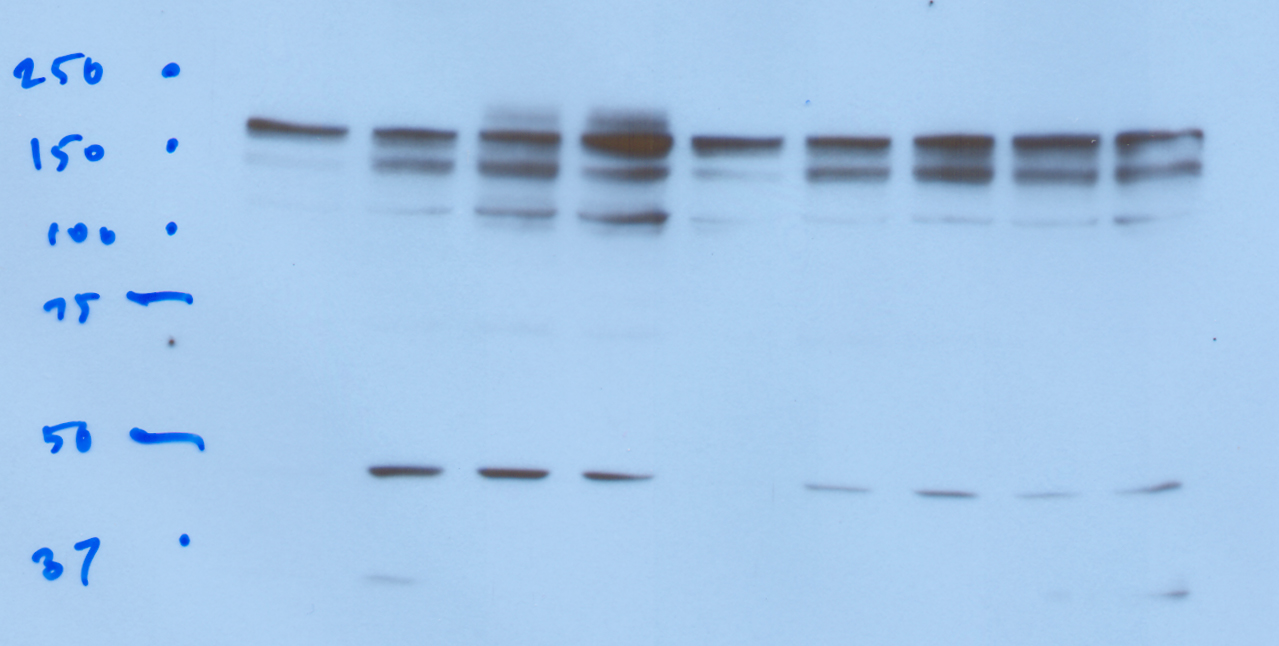

Supplement: Figure 2—figure supplement 2—source data 2. [file elife-96085-fig2-figsupp2-data2.zip › Figure 2-figure supplement 2-Source Data 2 - Raw unedited gels for Figure 2-figure supplement 2/Figure 2-figure supplement 2C_Middle_pY284_light.tiff]

Full unedited gels for Figure 4A. The red box shows the image used in the manuscript.

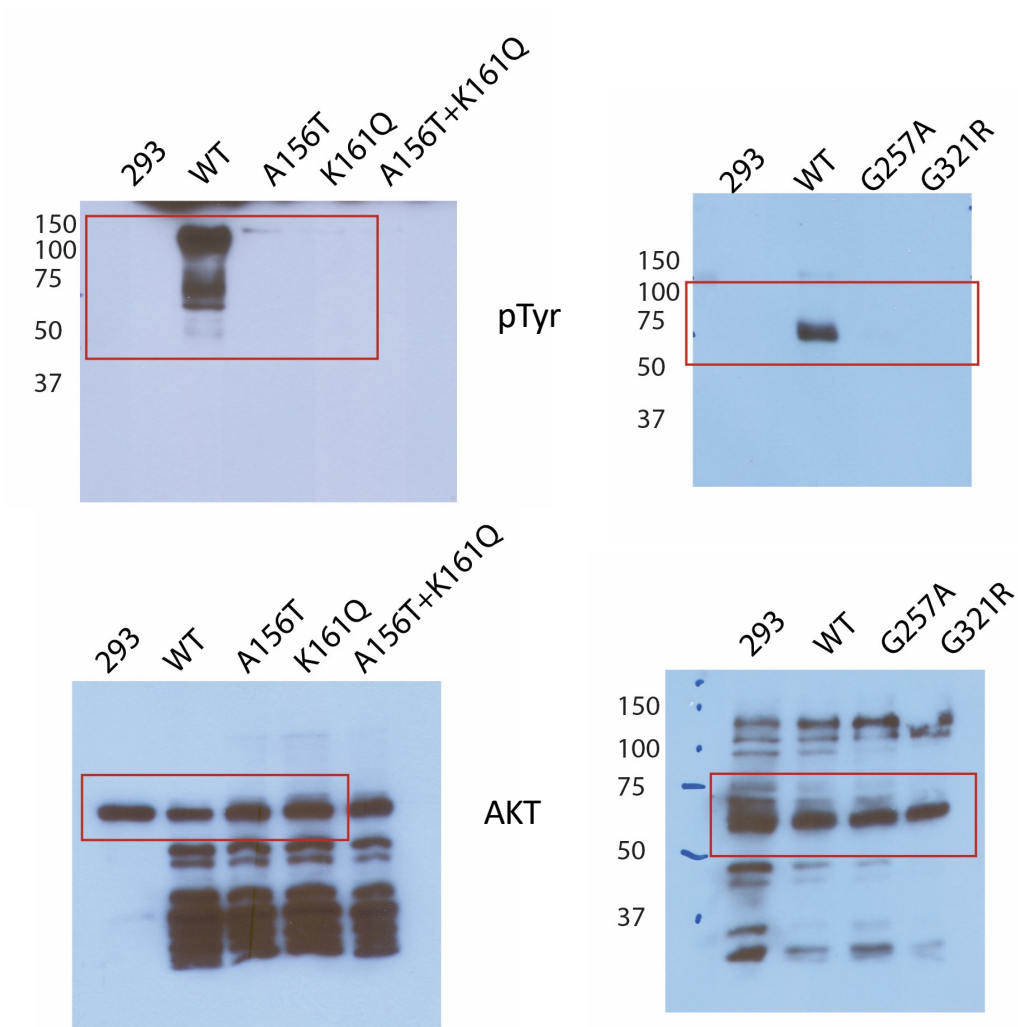

Supplement: Figure 4—source data 6. [file elife-96085-fig4-data6.zip › Figure 4-Source Data 6 - Uncropped and labelled gels for Figure 4/Figure 4 - Uncropped and labelled gels - Related to Figure 4A.pdf]

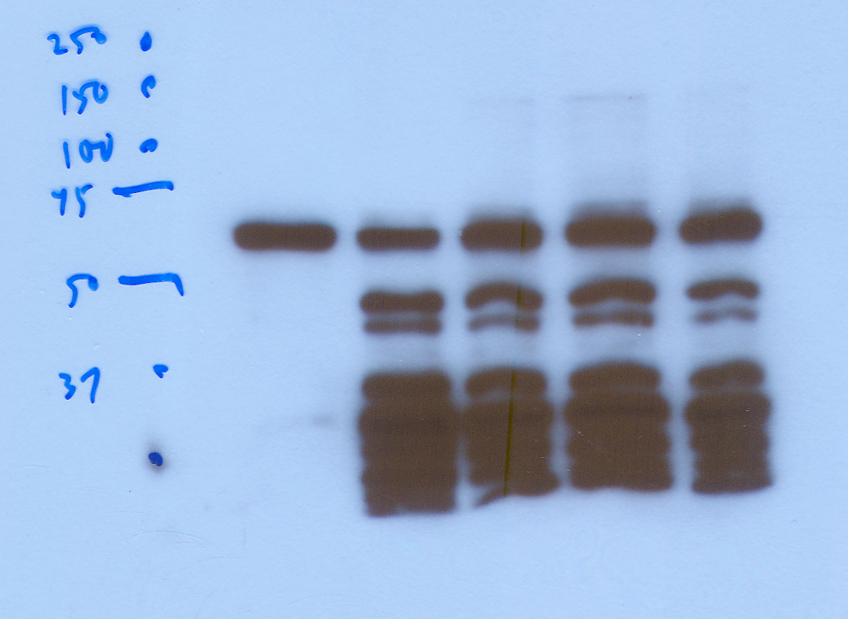

Supplement: Figure 4—source data 7. [file elife-96085-fig4-data7.zip › Figure 4-Source Data 7 - Raw unedited gels for Figure 4/Fig4A_Left_Bottom.tiff]

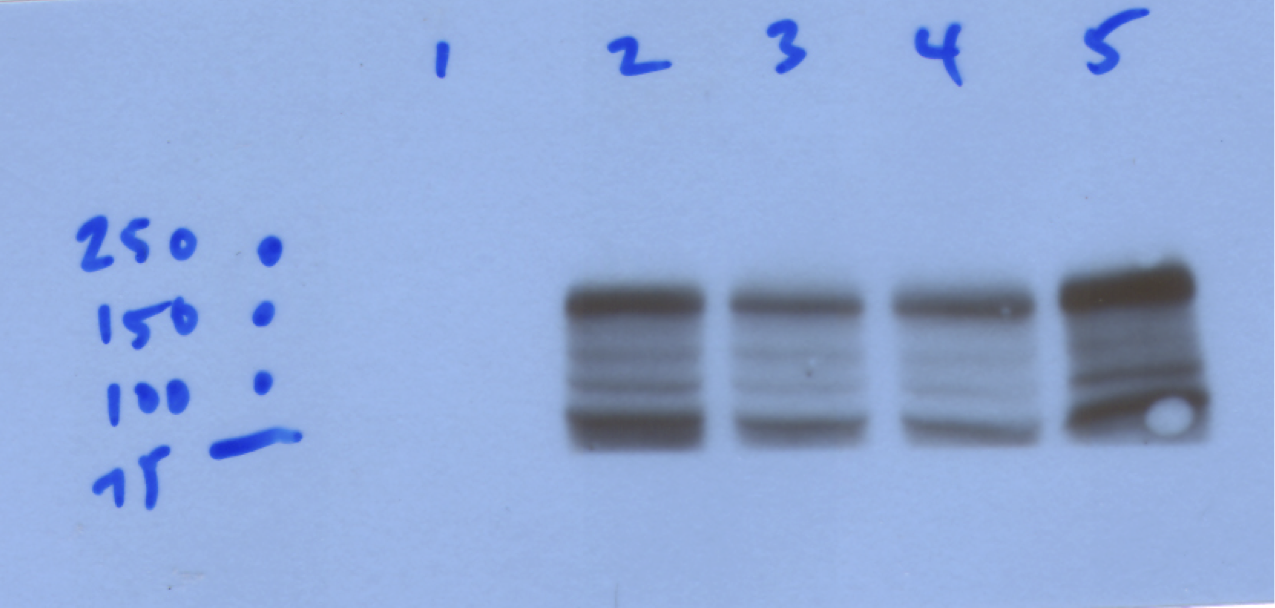

Supplement: Figure 4—source data 7. [file elife-96085-fig4-data7.zip › Figure 4-Source Data 7 - Raw unedited gels for Figure 4/Fig4C_Left_FLAG.tiff]

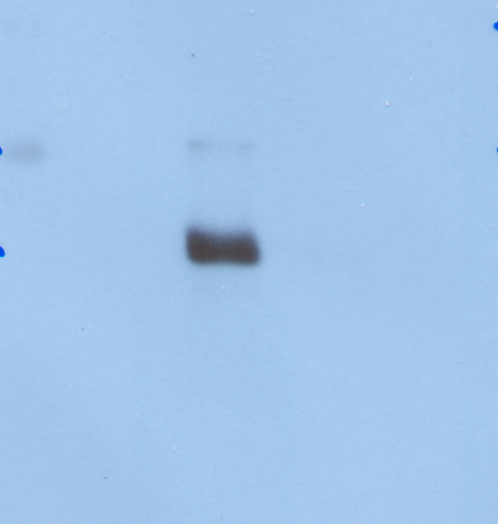

Supplement: Figure 4—source data 7. [file elife-96085-fig4-data7.zip › Figure 4-Source Data 7 - Raw unedited gels for Figure 4/Fig4A_Right_top.tiff]

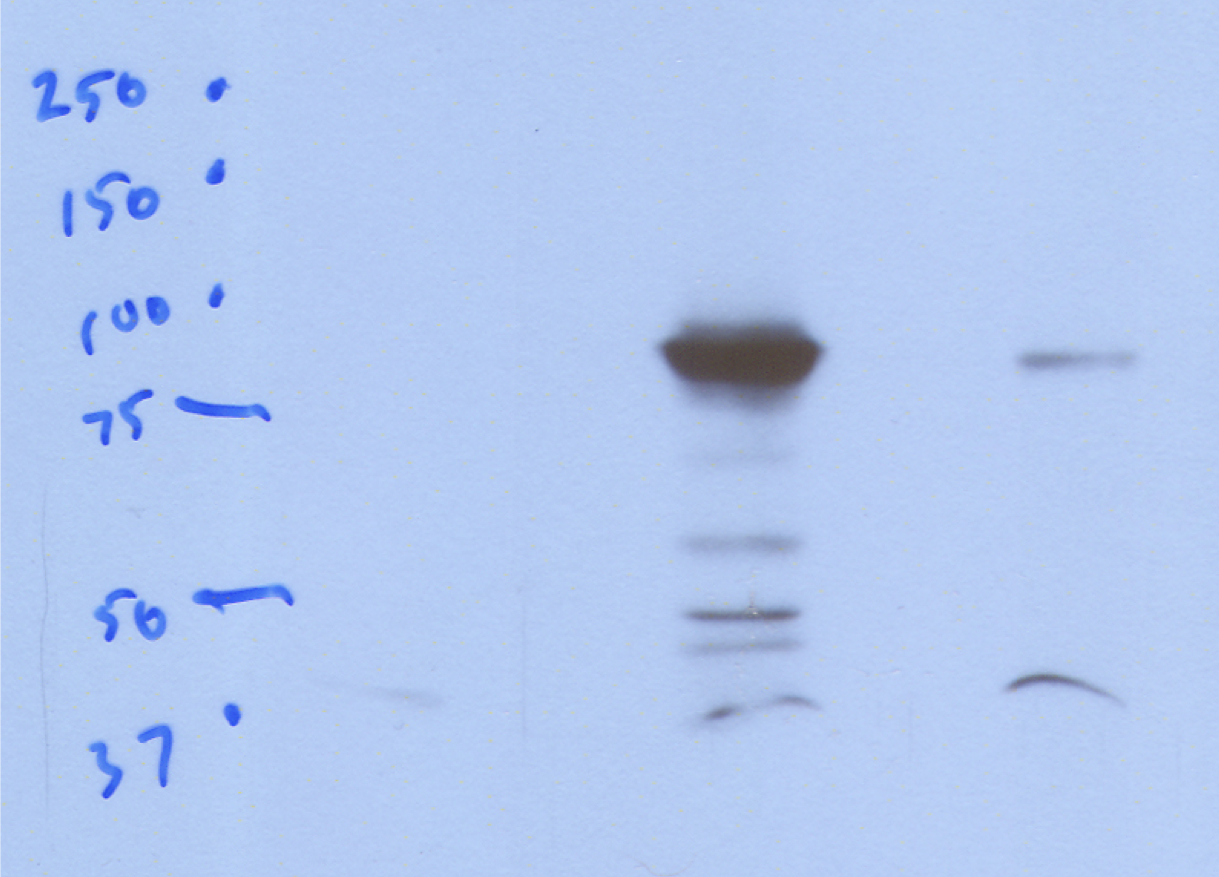

Supplement: Figure 4—source data 7. [file elife-96085-fig4-data7.zip › Figure 4-Source Data 7 - Raw unedited gels for Figure 4/Fig4B_Left_pY705.tiff]

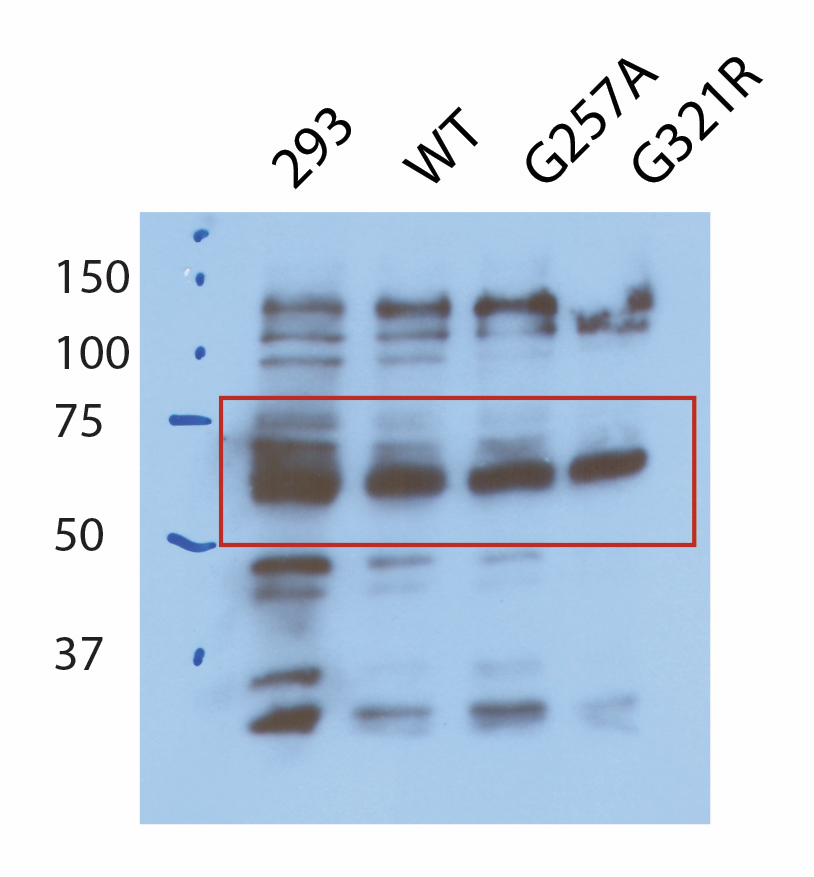

Supplement: Figure 4—source data 7. [file elife-96085-fig4-data7.zip › Figure 4-Source Data 7 - Raw unedited gels for Figure 4/Fig4A_Right_Bottom.tiff]

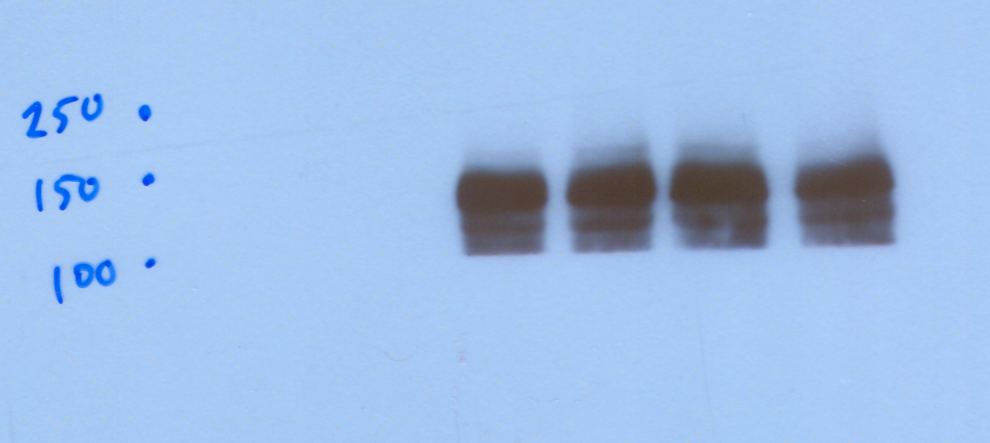

Supplement: Figure 4—source data 7. [file elife-96085-fig4-data7.zip › Figure 4-Source Data 7 - Raw unedited gels for Figure 4/Fig4B_Left_FLAG.tiff]

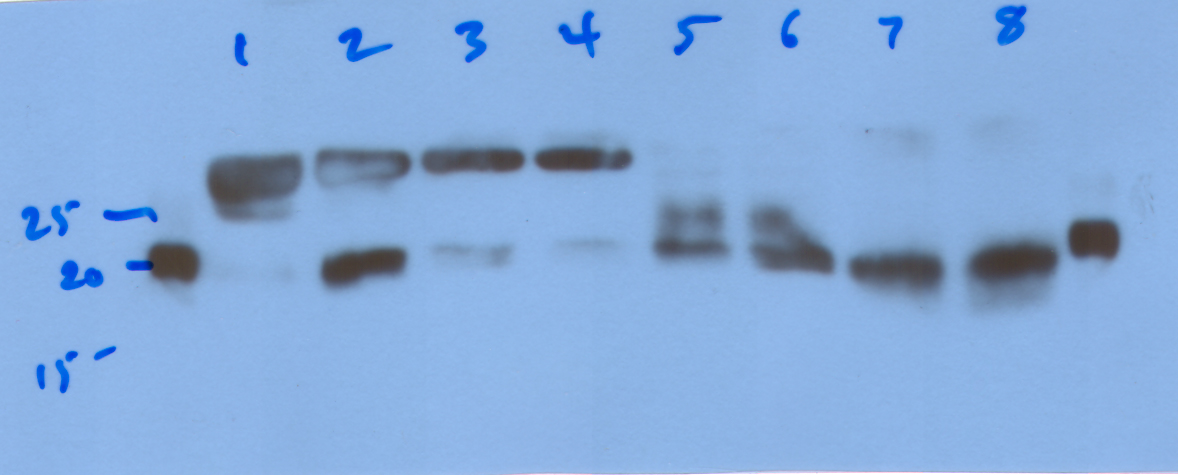

Supplement: Figure 4—source data 7. [file elife-96085-fig4-data7.zip › Figure 4-Source Data 7 - Raw unedited gels for Figure 4/Fig4C_right_Rac1pulldown.tiff]

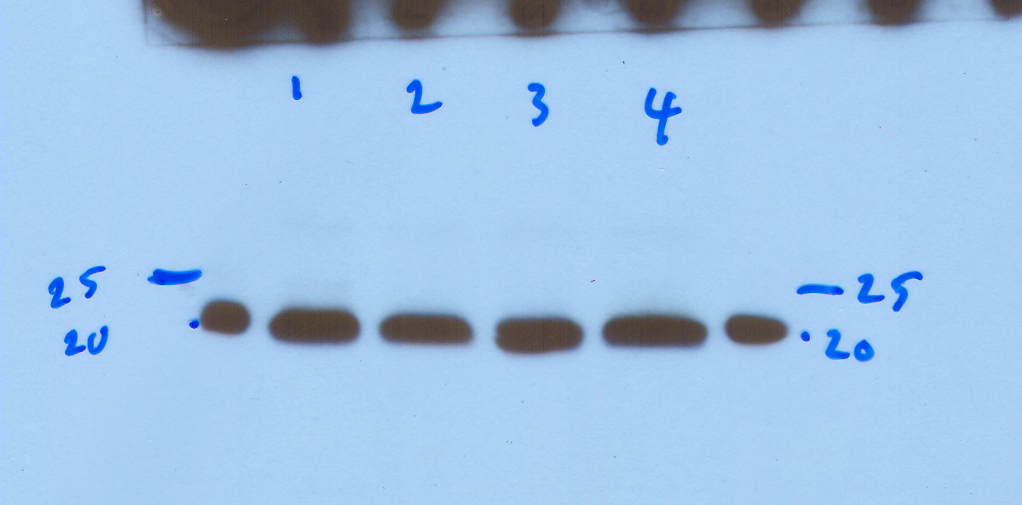

Supplement: Figure 4—source data 7. [file elife-96085-fig4-data7.zip › Figure 4-Source Data 7 - Raw unedited gels for Figure 4/Fig4C_Right_Rac1.tiff]

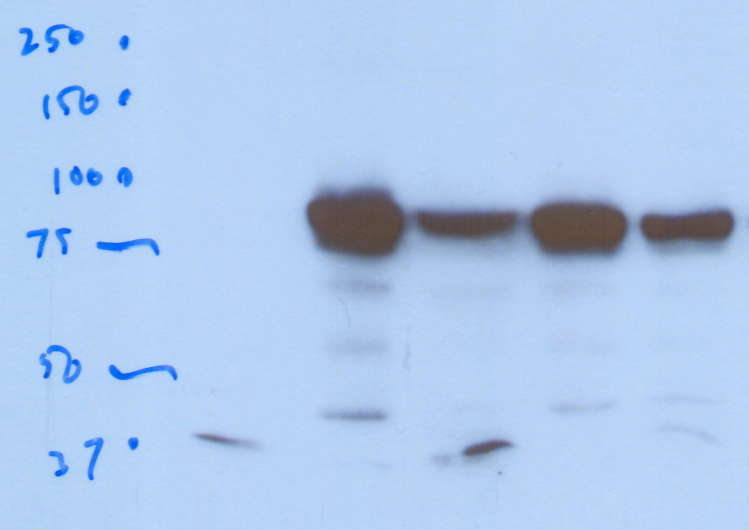

Supplement: Figure 4—source data 7. [file elife-96085-fig4-data7.zip › Figure 4-Source Data 7 - Raw unedited gels for Figure 4/Fig4B_Left_STAT3.tiff]

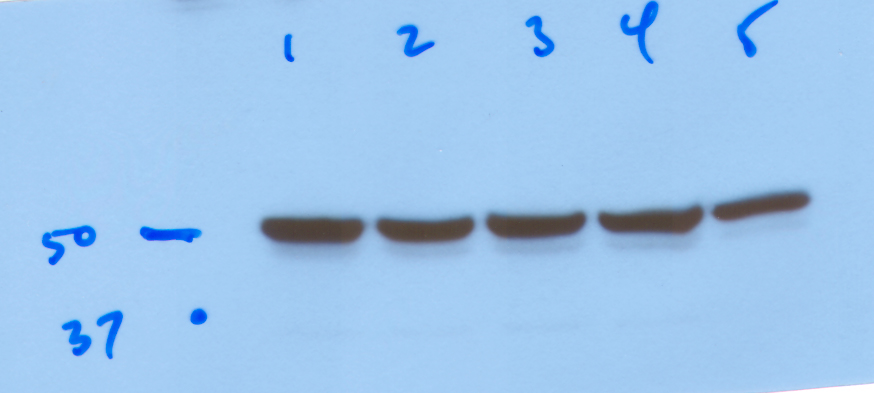

Supplement: Figure 4—source data 7. [file elife-96085-fig4-data7.zip › Figure 4-Source Data 7 - Raw unedited gels for Figure 4/Fig4C_Left_tubulin.tiff]

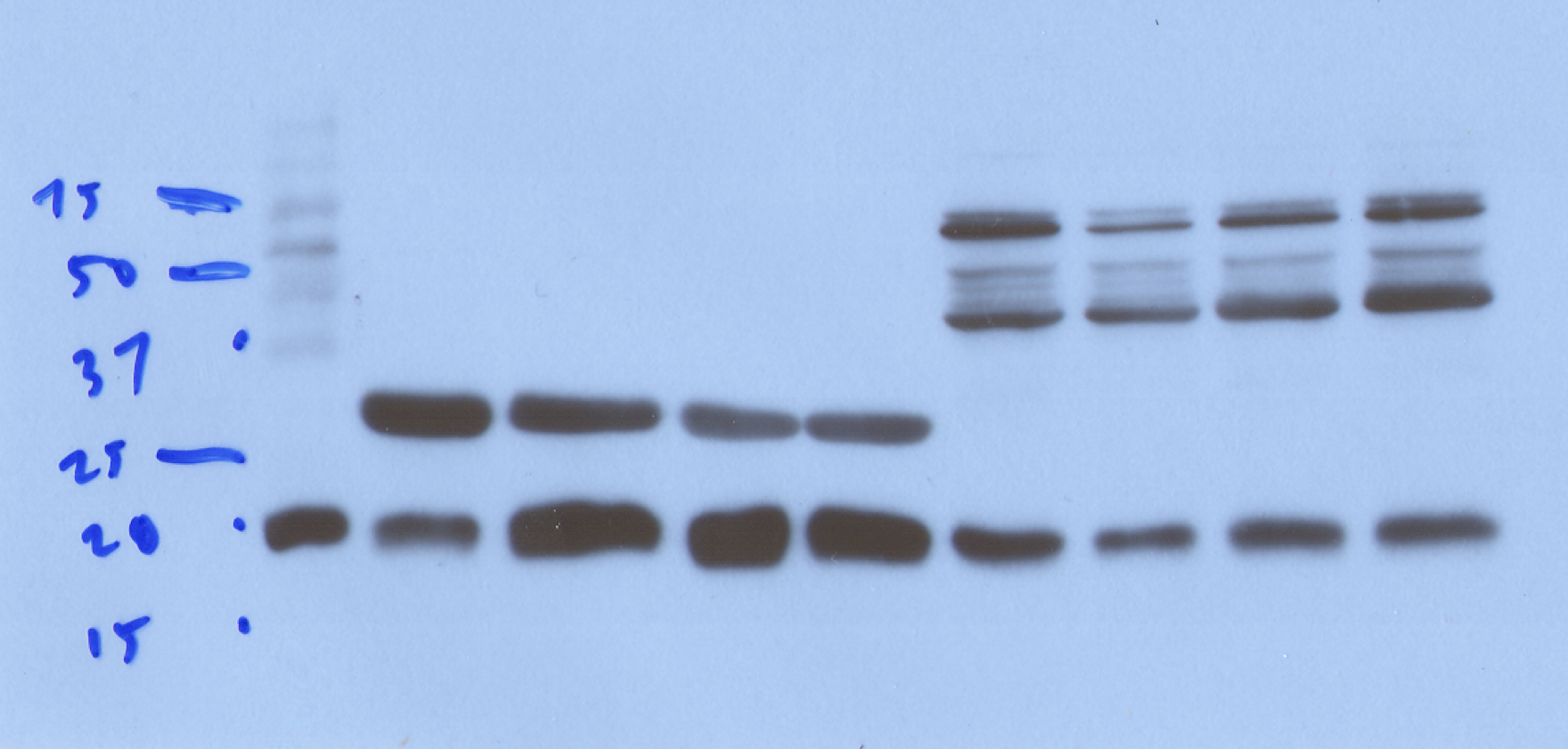

Supplement: Figure 4—source data 7. [file elife-96085-fig4-data7.zip › Figure 4-Source Data 7 - Raw unedited gels for Figure 4/Fig4C_Left_Rac1.tiff]

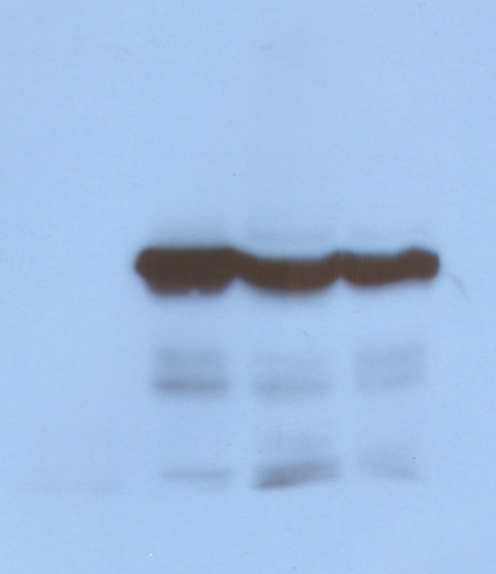

Supplement: Figure 4—source data 7. [file elife-96085-fig4-data7.zip › Figure 4-Source Data 7 - Raw unedited gels for Figure 4/Fig4B_Right_FLAG.tiff]

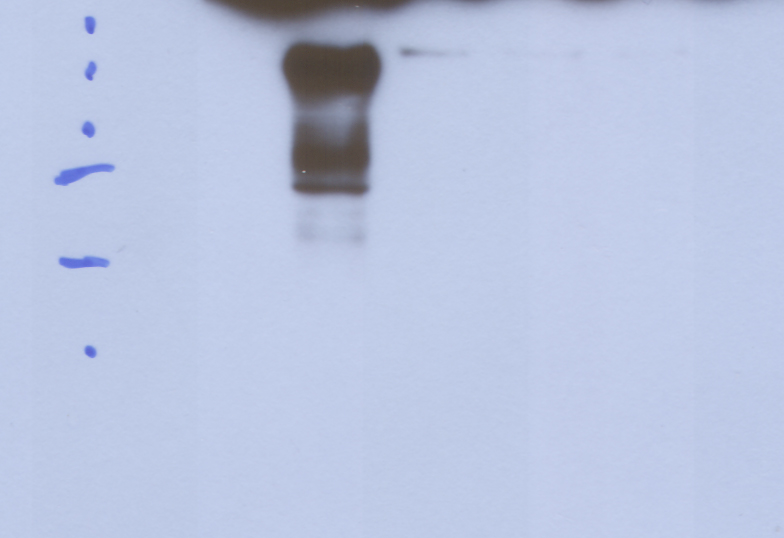

Supplement: Figure 4—source data 7. [file elife-96085-fig4-data7.zip › Figure 4-Source Data 7 - Raw unedited gels for Figure 4/Fig4A_Left_Top.tiff]

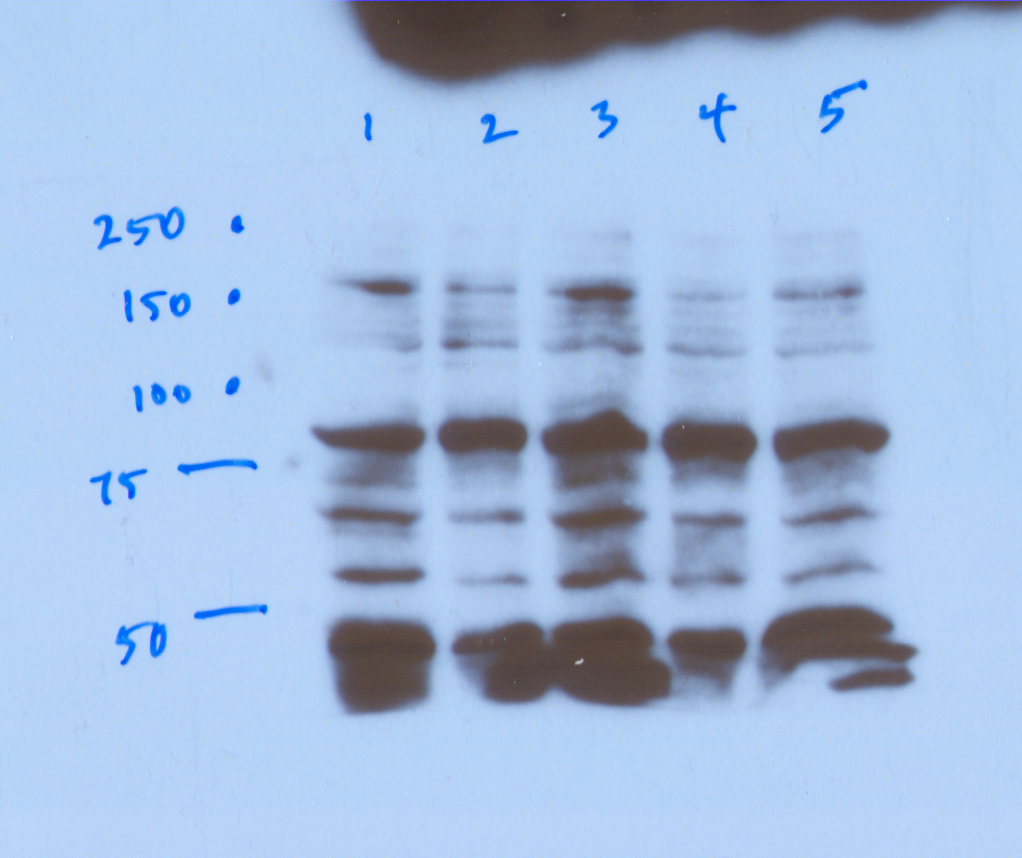

Supplement: Figure 4—source data 7. [file elife-96085-fig4-data7.zip › Figure 4-Source Data 7 - Raw unedited gels for Figure 4/Fig4B_Right_STAT3.tiff]

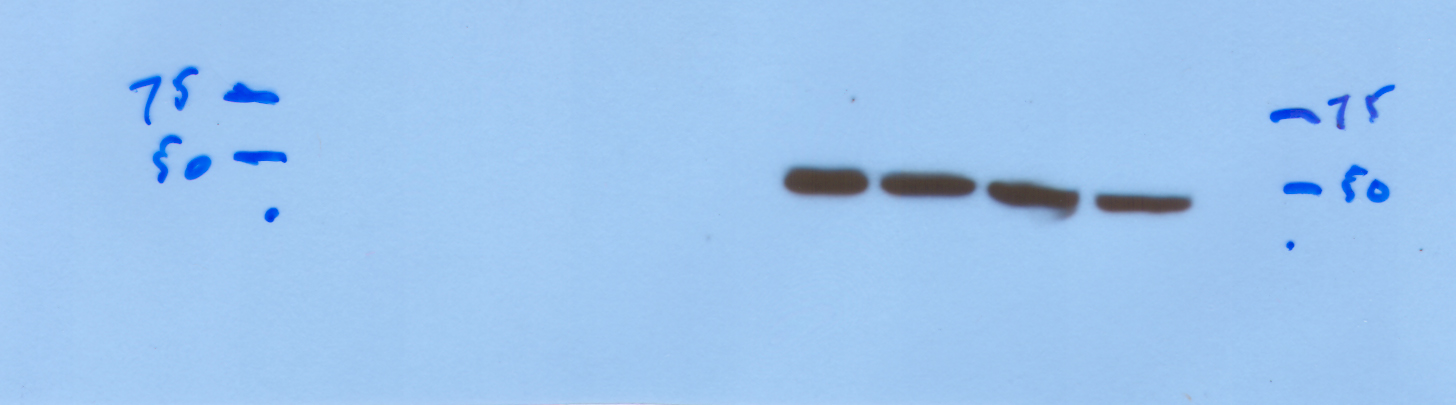

Supplement: Figure 4—source data 7. [file elife-96085-fig4-data7.zip › Figure 4-Source Data 7 - Raw unedited gels for Figure 4/Fig4C_Right_tubulin.tiff]

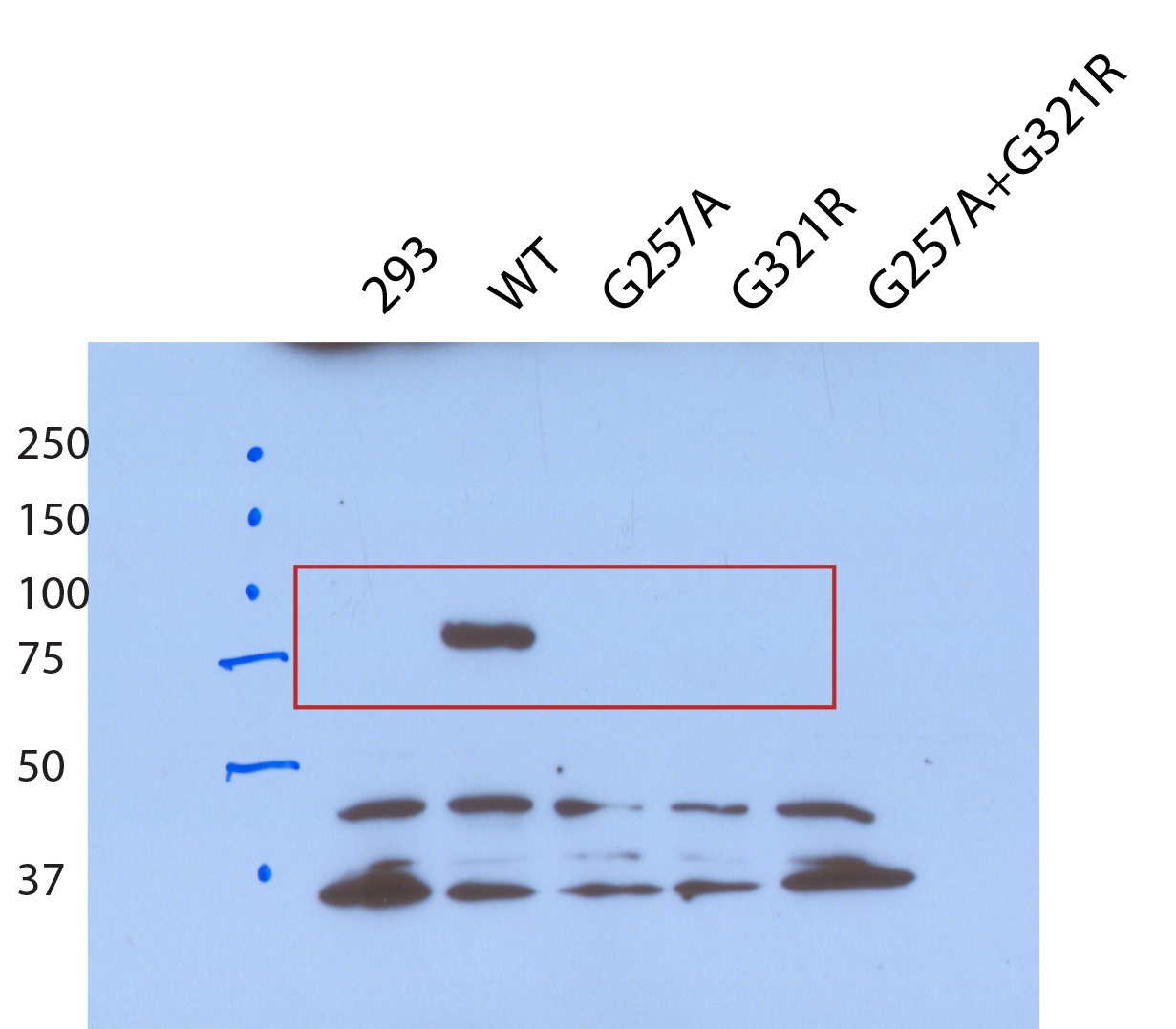

Supplement: Figure 4—source data 7. [file elife-96085-fig4-data7.zip › Figure 4-Source Data 7 - Raw unedited gels for Figure 4/Fig4B_Right_pY705.tiff]

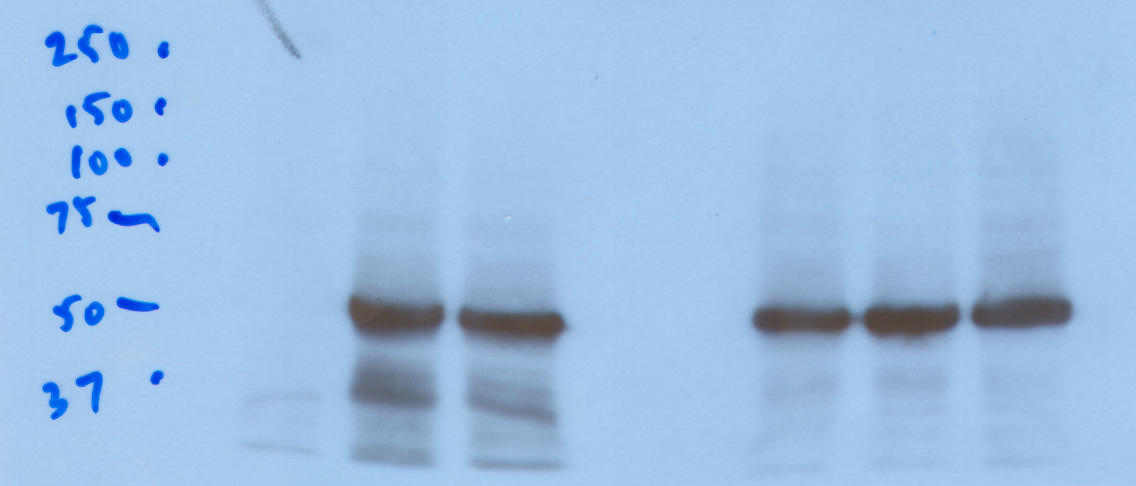

Supplement: Figure 4—source data 7. [file elife-96085-fig4-data7.zip › Figure 4-Source Data 7 - Raw unedited gels for Figure 4/Fig4C_Right_FLAG.tiff]

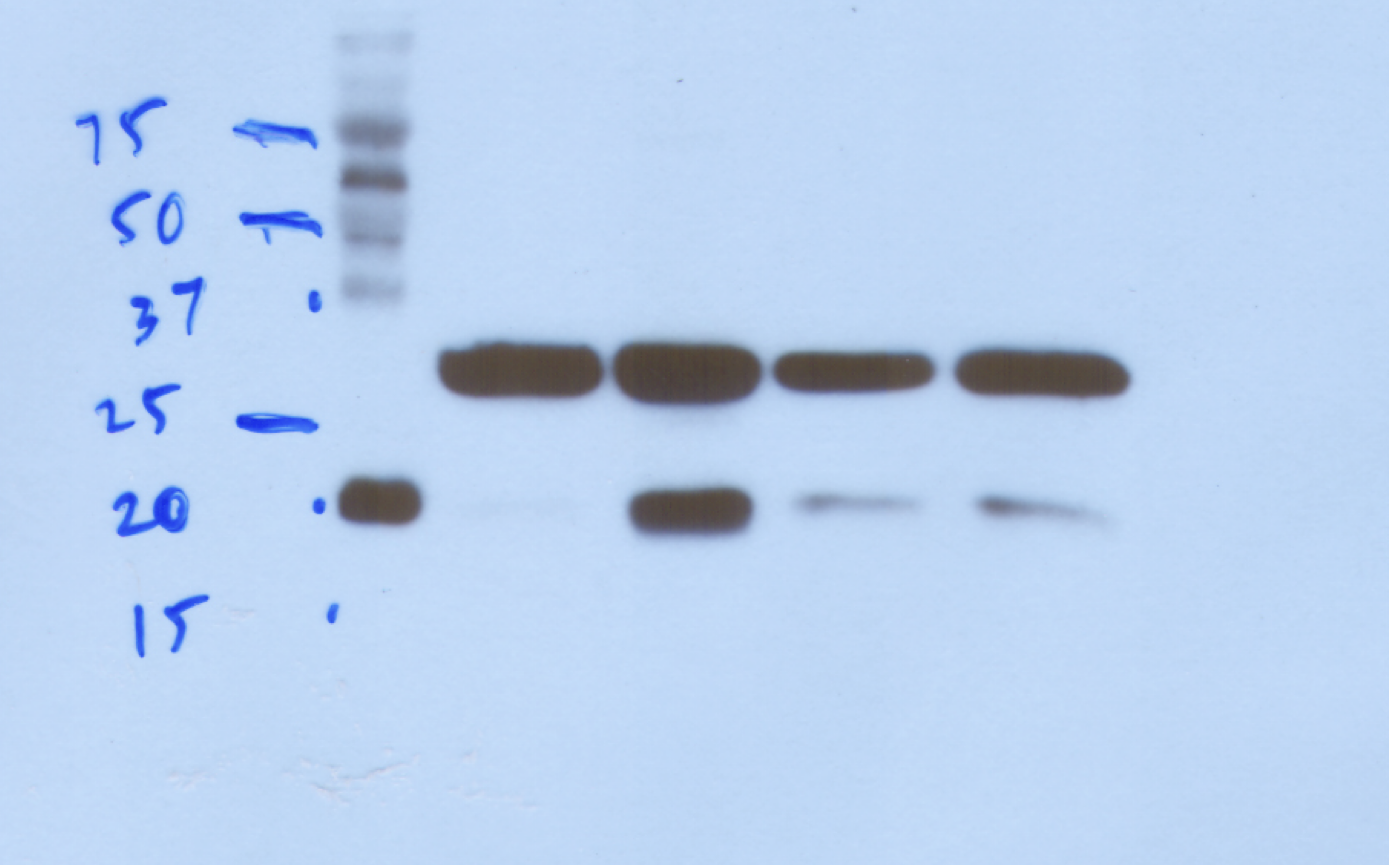

Supplement: Figure 4—source data 7. [file elife-96085-fig4-data7.zip › Figure 4-Source Data 7 - Raw unedited gels for Figure 4/Fig4C_Left_Rac1pulldown.tiff]
