# Supplementary material for: ACK1 and BRK non-receptor tyrosine kinase deficiencies are associated with familial systemic lupus and involved in efferocytosis
Source: eLife. 2024 Nov 21;13:RP96085. doi: 10.7554/eLife.96085 (PMC11581429; doi:10.7554/eLife.96085)
Supplement: Figure 2—figure supplement 2—source data 1. [file elife-96085-fig2-figsupp2-data1.zip › Figure 2-figure supplement 2 - Uncropped and labelled gels - Related to Figure 2-figure supplement 2C.pdf]

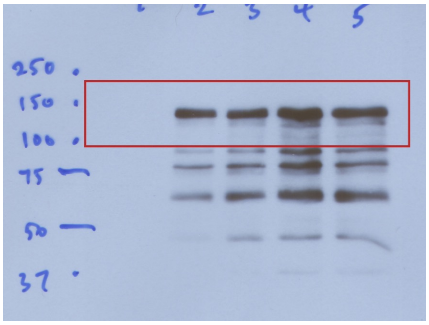

Full unedited gel for Figure 2-figure supplement 2C (Left). The red box shows the image used in the manuscript.

293 WT R99W R877H R1038H

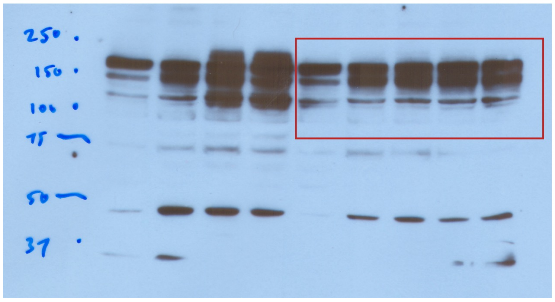

pY284 (dark)

293 WT R99W R877H R1038H

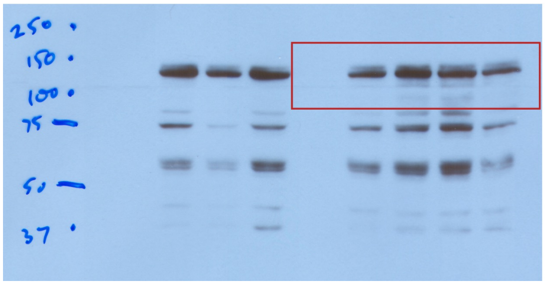

Flag

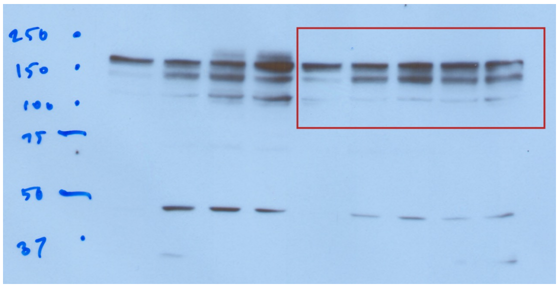

pY284 (light)

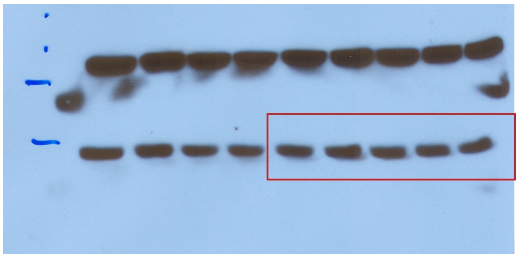

tubulin

Full unedited gel for Figure 2-figure supplement 2C (middle). The red box shows the image used in the manuscript.

293 WT V890M

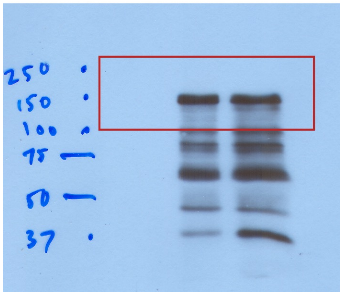

Full unedited gel for Figure 2-figure supplement 2C (right). The red box shows the image used in the manuscript.
