# Supplementary material for: ACK1 and BRK non-receptor tyrosine kinase deficiencies are associated with familial systemic lupus and involved in efferocytosis
Source: eLife. 2024 Nov 21;13:RP96085. doi: 10.7554/eLife.96085 (PMC11581429; doi:10.7554/eLife.96085)

Full unedited gels for Figure 4B (left). The red box shows the image used in the manuscript.

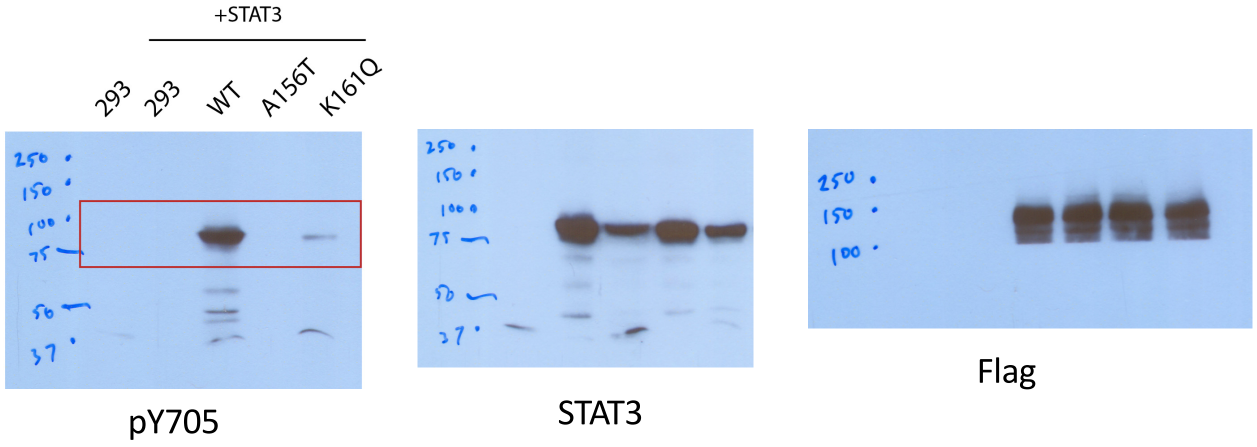

Full unedited gels for Figure 4B(right). The red box shows the image used in the manuscript.

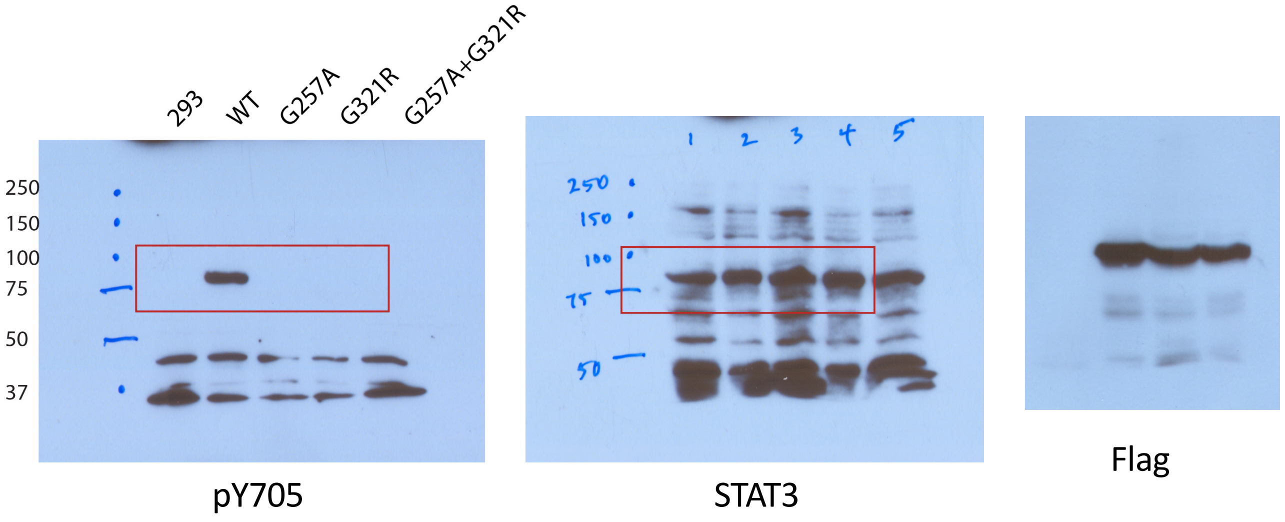

Supplement: Figure 4—source data 6. [file elife-96085-fig4-data6.zip › Figure 4-Source Data 6 - Uncropped and labelled gels for Figure 4/Figure 4 - Uncropped and labelled gels - Related to Figure 4B.pdf]
